# Modulating Immunogenicity and Reactogenicity in mRNA-Lipid Nanoparticle Vaccines through Lipid Component Optimization

**DOI:** 10.1021/acsnano.5c10648

**Published:** 2025-07-23

**Authors:** Yoshino Kawaguchi, Mari Kimura, Tatsuya Karaki, Hiroki Tanaka, Chikako Ono, Tatsuhiro Ishida, Yoshiharu Matsuura, Toshiro Hirai, Hidetaka Akita, Taro Shimizu, Yasuo Yoshioka

**Affiliations:** † Vaccine Creation Group, BIKEN Innovative Vaccine Research Alliance Laboratories, Research Institute for Microbial Diseases, The University of Osaka, 3-1 Yamadaoka, Suita, Osaka 565-0871, Japan; ‡ Department of Pharmacokinetics and Biopharmaceutics, Graduate School of Biomedical Sciences, Tokushima University, 1-78-1 Sho-machi, Tokushima, Tokushima 770-8505, Japan; § 13013 The Research Foundation for Microbial Diseases of Osaka University, 3-1 Yamadaoka, Suita, Osaka 565-0871, Japan; ∥ Laboratory of DDS Design and Drug Disposition, Graduate School of Pharmaceutical Sciences, 13101 Tohoku University, 6-3 Aoba, Aramaki, Aoba-ku, Sendai, Miyagi 980-8578, Japan; ⊥ Center for Advanced Modalities and DDS, The University of Osaka, 3-1 Yamadaoka, Suita, Osaka 565-0871, Japan; # Laboratory of Virus Control, Research Institute for Microbial Diseases, The University of Osaka, 3-1 Yamadaoka, Suita, Osaka 565-0871, Japan; ∇ Center for Infectious Disease Education and Research, The University of Osaka, 3-1 Yamadaoka, Suita, Osaka 565-0871, Japan; ○ Vaccine Creation Group, BIKEN Innovative Vaccine Research Alliance Laboratories, Institute for Open and Transdisciplinary Research Initiatives, The University of Osaka, 3-1 Yamadaoka, Suita, Osaka 565-0871, Japan; ◆ Laboratory of Nano-Design for Innovative Drug Development, Graduate School of Pharmaceutical Sciences, The University of Osaka, 1-6 Yamadaoka, Suita, Osaka 565-0871, Japan; ¶ Global Center for Medical Engineering and Informatics, The University of Osaka, 3-1 Yamadaoka, Suita, Osaka 565-0871, Japan

**Keywords:** cholesterol, lipid nanoparticle, mRNA vaccine, PEG-lipids, phospholipids

## Abstract

Messenger RNA (mRNA) vaccines effectively induce antibody production and T cell responses. However, adverse reactions, such as fatigue and fever, following administration remain a key challenge. To modulate the immunogenicity and reactogenicity of mRNA vaccines, the optimization of lipid nanoparticle (LNP) formulations has been attempted, particularly by screening ionizable lipids. In contrast, the potential impact of modifying other LNP components—poly­(ethylene glycol) (PEG)-lipids, cholesterol, and phospholipids—on overall vaccine effects and adverse reactions remains underexplored. Here, we prepared mRNA-LNP formulations with altered structures and molar ratios of these components to assess their effects on *in vivo* protein expression, as well as on the induction of antigen-specific immune responses and adverse reactions. Reducing the PEG chain length and molar ratio of PEG-lipids increased antigen-specific antibody and CD8^+^ T cell responses. LNPs with cholesterol substituted by plant sterols, or LNPs with phospholipids replaced by those with different head and tail group structures, induced antigen-specific antibody and CD8^+^ T cell responses comparable to the control formulation. Alternately, these LNPs significantly reduced inflammatory cytokine production and adverse reactions, including fever, compared with the control LNPs. Finally, correlation analysis revealed a positive association between protein expression in specific organs and the magnitude of immune responses and adverse reactions. These findings demonstrate that modifying PEG-lipids, cholesterol, and phospholipids is beneficial for modulating the immunogenicity and reactogenicity of the mRNA-LNP vaccine.

## Graphical abstract

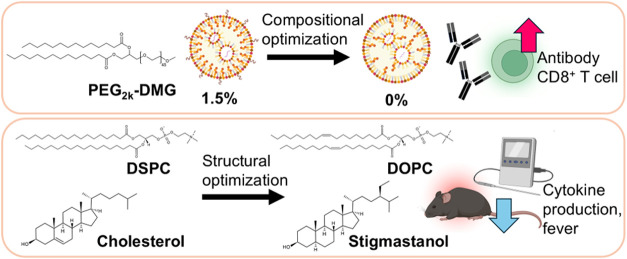

The coronavirus disease 2019 (COVID-19) pandemic has accelerated the clinical application of messenger RNA (mRNA) vaccines, leading to the first Food and Drug Administration (FDA) approval of mRNA vaccines against SARS-CoV-2: BNT162b2 (BioNTech/Pfizer) and mRNA-1273 (Moderna). These vaccines induce robust humoral and cellular immune responses against SARS-CoV-2, and one study showed 94% effectiveness in preventing symptomatic SARS-CoV-2 infection after a second dose under real-world conditions.^1^ In addition, the development of mRNA vaccines for various infectious diseases and cancer treatments is also progressing,^2,3^ as exemplified by the recent FDA approval of an mRNA vaccine for respiratory syncytial virus (RSV).^4^ However, despite their high efficacy, mRNA vaccines frequently cause adverse reactions, such as pain at the injection site, fever, and fatigue,^5–7^ contributing to vaccine hesitancy among the public.^8,9^ Addressing these adverse reactions is critical to improving public acceptance.

A key factor behind the immunogenicity and reactogenicity of mRNA vaccines lies in their formulation. Currently approved mRNA vaccines encapsulate antigen-encoding mRNA in lipid nanoparticles (mRNA-LNP).^10^ LNPs serve multiple critical roles: they protect mRNA from degradation,^11^ facilitate efficient cellular uptake,^12^ and deliver mRNA into the cytoplasm.^13^ Furthermore, LNPs activate innate immunity and serve as adjuvants to elicit strong antigen-specific immune responses.^14,15^ However, they can also induce excessive inflammatory responses, causing local and systemic adverse reactions.^16–18^ Therefore, optimizing LNP formulations is necessary to minimize reactogenicity and reduce adverse reactions while maintaining the ability of the vaccine to provoke a robust antigen-specific immune response.

LNPs comprise four main components: ionizable lipids, poly­(ethylene glycol) (PEG)-conjugated lipids (PEG-lipids), cholesterol, and phospholipids.^10,19^ Among these components, ionizable lipids, which become positively charged at pH levels below their p*K*_a_, play a pivotal role in encapsulating negatively charged mRNA payload and associating with negatively charged endosomal membranes.^20,21^ Ionizable lipids are recognized by the innate immune system,^22^ leading to both adjuvant activity and adverse reactions. Owing to their important roles in mRNA delivery and immune responses, ionizable lipids have been the primary focus for optimizing LNP formulations. Among the numerous candidate ionizable lipids with distinct chemical structures, those capable of maintaining LNPs’ immune induction activity while exhibiting minimal pro-inflammatory properties have been investigated.^23,24^ Notably, the mRNA vaccines developed by Pfizer-BioNTech and Moderna utilize ionizable lipids that are independently optimized for their mRNA vaccines.

On the other hand, in previous efforts to optimize LNP formulations for the development of vaccines with both enhanced immunogenicity and reduced pro-inflammatory properties, relatively little attention has been paid to lipid components other than ionizable lipids—PEG-lipids, cholesterol, and phospholipids. However, it has been reported that modifications to these lipid components can also significantly influence the physicochemical properties of the LNPs and efficiency of mRNA delivery. Furthermore, some analogs of these lipids reportedly exhibit intrinsic biological activities, including anti-inflammatory effects. Therefore, modifying these lipid components may also enable the development of low-inflammatory LNPs, potentially leading to the development of mRNA vaccines with reduced adverse reactions. First, PEG-lipid forms a hydrophilic steric barrier that prevents LNP aggregation in the formulation.^25^ In clinically approved mRNA vaccines, PEG with an average molecular weight of 2000 conjugated to dimyristoyl glycerol (DMG) is incorporated into LNP. The effect of PEG-lipid modifications on particle stability and drug delivery has been extensively evaluated for decades using liposomal and siRNA-LNP formulations.^26,27^ Increasing the PEG chain length or incorporation ratio of PEG-lipids enhances the stability and circulation time of intravenous PEGylated particles.^28,29^ In contrast, PEGylation inhibits the cellular uptake of particles, thereby decreasing delivery to target cells.^30,31^ Similarly, altering the PEG chain length and molar ratio of PEG-lipids in mRNA-LNPs affects transfection efficiency *in vitro* and *in vivo*.^32–35^ Based on these reports, optimizing the PEG length and molar ratio of PEG-lipids may minimize reactogenicity while maintaining the vaccine immunogenicity. Second, cholesterol plays a crucial role in regulating the rigidity and stability of lipid membranes. In most previous LNP studies, as well as in clinically used mRNA vaccines, natural cholesterol has been employed as an excipient. Recent studies have suggested that replacing cholesterol with plant sterols, such as β-sitosterol, campesterol, fucosterol, and stigmastanol, enhances *in vitro* transfection efficiency.^36^ Furthermore, several *in vitro* and *in vivo* studies have reported that plant sterols, such as β-sitosterol and campesterol, exhibit direct anti-inflammatory effects as part of their biological activity. These findings suggest that optimization of the cholesterol species within LNPs may enhance protein expression efficiency and reduce reactogenicity in mRNA-LNP vaccines. However, the *in vivo* utility of mRNA-LNPs containing these plant-derived sterols remains unclear. Finally, phospholipids stabilize the particles and aid in mRNA solubilization. In clinical vaccines, distearoylphosphatidylcholine (DSPC) is the phospholipid used in LNP formulations. However, altering the type of phospholipid affects the membrane fluidity and charge of particles. Consequently, this modification influences transfection efficiency *in vitro* and organ distribution of LNPs following intravenous administration.^33,37^ Furthermore, fatty acid moieties within phospholipid molecules are known to possess distinct pro-inflammatory or anti-inflammatory activities. Specifically, saturated fatty acids incorporated in phospholipids—such as stearic acid—are generally recognized as pro-inflammatory agents, whereas unsaturated fatty acids—such as oleic acid—reportedly exhibit anti-inflammatory effects.^38,39^ Therefore, the optimization of phospholipid species within LNPs may contribute to the reduction of reactogenicity in mRNA-LNP vaccines. These findings strongly suggest that the optimization of the composition ratios and selection of molecular species of PEG-lipids, cholesterol, and phospholipids may also be beneficial for the development of mRNA-LNP vaccines with minimized reactogenicity and maintained immunogenicity. However, the effects of the structural and compositional modifications of these three components within LNPs on *in vivo* protein expression, immune response induction, and adverse reactions following intramuscular administration have not been fully evaluated.

In this study, we prepared mRNA-LNPs with varying structures and compositions of PEG-lipids, cholesterol, and phospholipids, aiming to evaluate the impact of each component on *in vivo* protein expression, immune responses, and adverse reactions in mice following intramuscular administration as mRNA vaccines.

## Results

### Experimental Overview

This study evaluated the effects of the PEG-lipid chain length (Figures 2 and 3), PEG-lipid molar ratio (Figures 4 and 5), cholesterol structures (Figures 6 and 7), and phospholipid structures (Figures 8 and 9) on *in vivo* protein expression, antigen-specific immune responses, and adverse reactions induced by mRNA-LNPs. We used SM-102 as an ionizable lipid and LNP comprising SM-102/DSPC/Cholesterol/PEG_2k_-DMG (50:10:38.5:1.5 molar ratio) as the control LNP. To minimize innate immune stimulation, all mRNA was modified with *N* ^1^-methyl-pseudouridine, 5′-capped with a cap1 analog, and purified to remove double-stranded RNA (dsRNA) using a cellulose column. As illustrated in Figure 1A, *in vivo* protein expression was evaluated by administering LNP-encapsulating firefly luciferase (FLuc)-encoding mRNA (FLuc-LNP) into the tibialis anterior (TA) muscle of mice and measuring FLuc activity at the injection site (TA), draining lymph nodes (dLN, inguinal lymph nodes), spleen, and liver at 6 and 24 h postinjection. For immune response evaluation (Figure 1B), mice were vaccinated with LNP encapsulating mRNA encoding the spike (S) protein of SARS-CoV-2 of the Wuhan-Hu-1 strain (S-LNP) twice at a 3-week interval (prime and boost, 1 μg mRNA/mouse). The S protein is crucial for viral entry and is the main target of SARS-CoV-2 vaccines. Phosphate buffer saline (PBS) was used as a negative control. Blood levels of S-specific IgG were measured on days 14 and 28, whereas IgG subclasses (IgG1 and IgG2b) and neutralizing antibody titers were evaluated on day 28. Neutralizing antibody titers were measured with a pseudotyped virus displaying S protein of SARS-CoV-2, and the highest dilution to achieve infection of ≤ 90% or ≤ 99% was considered the NT_90_ and NT_99_ titer for the sample, respectively. S-specific CD8^+^ T cell numbers in the blood were quantified on days 10 and 28 using flow cytometry with the H-2k^b^ S_539–546_ tetramer (Stet, gating method is shown in Figure S1). Finally, as illustrated in Figure 1C, adverse reactions were evaluated by measuring blood inflammatory cytokine levels, rectal temperature 6 h after a single administration of high-dose S-LNPs (20 μg mRNA/mouse), and body weight changes 24 h postadministration.

**Figure 1:**
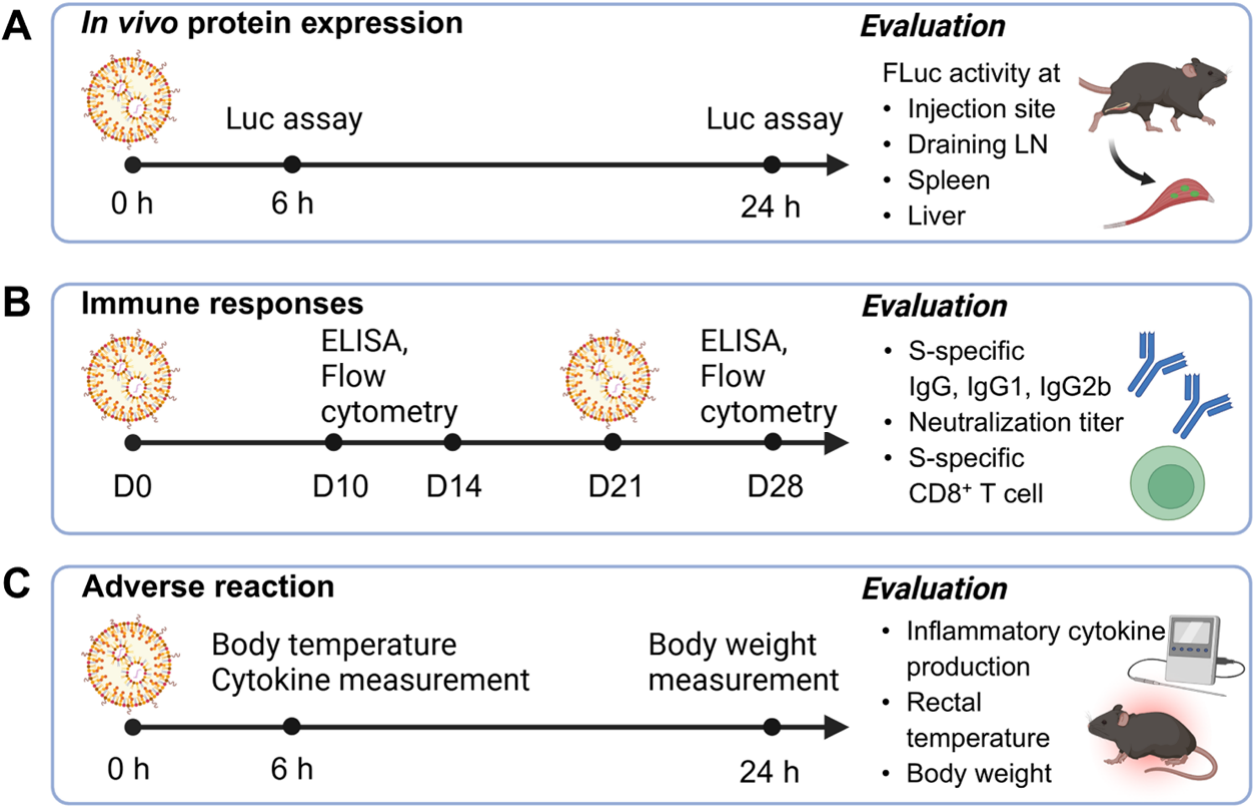
Experimental overview. *In vivo* protein expression, immune responses, and adverse reactions were compared between modified LNPs and control LNP. (A) *In vivo* protein expression was evaluated by administering LNP encapsulating FLuc-mRNA (FLuc-LNP) into the TA of C57BL/6J mice (1 μg mRNA/mouse) and measuring FLuc activity at the TA, dLN, spleen, and liver at 6 and 24 h postinjection. (B) Immune responses were evaluated by vaccinating C57BL/6J mice with LNPs encapsulating S-mRNA (S-LNP) twice at 3-week intervals (1 μg mRNA/mouse). S-specific IgG levels in the blood were quantified on days 14 and 28. S-specific IgG1 and IgG2b levels and neutralizing antibody titers were also evaluated at day 28. S-specific CD8^+^ T cell numbers in the blood were quantified on days 10 and 28. (C) Adverse reactions were evaluated based on the blood levels of inflammatory cytokines and changes in rectal temperature measured 6 h after a single administration of S-LNP (20 μg mRNA/mouse) into C57BL/6J mice, as well as changes in body weight assessed 24 h postadministration.

### Effect of PEG Chain Length

First, we focused on PEG-lipid and evaluated the effect of PEG chain length on protein expression and immune responses. LNPs were formulated by replacing PEG_2k_-DMG with PEG-lipids containing shorter (average MW 1000, PEG_1k_-DMG) or longer (average MW 5,000, PEG_5k_-DMG) PEG chains while maintaining their molar ratio (PEG_1k_-LNP and PEG_5k_-LNP, respectively). The characteristics of these LNPs are presented in Figure 2A, where PEG_1k_-LNP had a significantly larger particle size than the control PEG_2k_-LNP. At 6 h postadministration of FLuc-LNPs, similar levels of FLuc expression were observed in the TA across all groups (Figure 2B). However, the PEG_1k_-LNP group showed increased expression in the spleen compared with the PEG_2k_-LNP group, whereas the PEG_5k_-LNP group exhibited decreased expression in both the dLN and spleen compared with the PEG_2k_-LNP group (Figure 2B). A similar trend was observed at 24 h postadministration; however, protein expression in the liver in the PEG_1k_-LNP group and in the TA in the PEG_5k_-LNP group was lower than that in the PEG_2k_-LNP group (Figure 2C).

**Figure 2:**
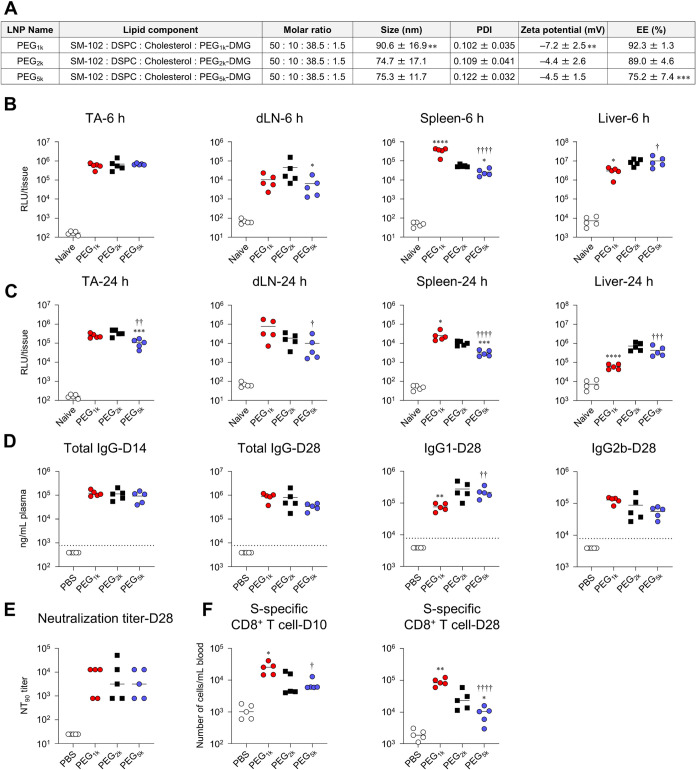
Effect of chain length of PEG on *in vivo* protein expression and immune responses induced by mRNA-LNPs. (A) Lipid composition and characteristics of LNPs with different PEG chain lengths. (B, C) Mice were intramuscularly administrated with FLuc-LNPs. FLuc activity in the homogenate of TA, dLN, spleen, and liver collected at (B) 6 h and (C) 24 h postadministration was measured as relative luminescence unit (RLU). The results for the naive groups in (B) and (C) are identical. (D-F) Mice were vaccinated intramuscularly with S-LNPs. (D) S-specific IgG in the plasma on day 14, S-specific IgG, IgG1, and IgG2b on day 28 were evaluated using ELISA. The dotted lines indicate the limit of detection. (E) Evaluation of neutralization activity of serum on day 28 against VSV-based pseudotyped viruses expressing the S protein of SARS-CoV-2 and determination of NT_90_. (F) S-specific CD8^+^ T cells in blood at days 10 and 28 were analyzed via flow cytometry. *P < 0.05, **P < 0.01, ***P < 0.001, ****P < 0.0001 vs PEG_2k_; ^†^ *P* < 0.05, ^††^ *P* < 0.01, ^†††^ *P* < 0.001, ^††††^ *P* < 0.0001 vs PEG_1k_ as determined by one-way ANOVA and Tukey’s multiple comparison test.

Next, we evaluated immune responses. Regardless of the PEG chain length, similar levels of S-specific IgG were induced in all groups after prime and boost vaccinations (Figure 2D). However, after the booster dose, S-specific IgG1 levels were significantly lower in the PEG_1k_-LNP group than in the PEG_2k_-LNP group, whereas no differences were observed between the groups for IgG2b levels (Figure 2D). The 90% and 99% neutralization titers (NT_90,_ NT_99_) in the sera of all the groups were comparable (Figure 2E, Figure S2A). After priming and boost, PEG_1k_-LNP induced a significantly greater number of S-specific CD8^+^ T cells compared with the PEG_2k_-LNP group (Figure 2F). Conversely, the PEG_5k_-LNP group exhibited significantly lower levels of induction of S-specific CD8^+^ T cells after the booster dose than the PEG_2k_-LNP group (Figure 2F).

We further quantified blood cytokine levels as markers of inflammation. The LNPs induced the production of various pro-inflammatory cytokines and chemokines (Figure 3A). For most cytokines and chemokines, such as type I IFN, IFN-γ, IL-6, IL-12p70, MCP-1, RANTES, and IP-10, the PEG_1k_-LNP group exhibited significantly higher levels than did the PEG_2k_-LNP group. In contrast, no significant differences were observed between the PEG_2k_-LNP and PEG_5k_-LNP groups (Figure 3A). In addition, a body temperature increase of approximately 1 °C was observed in the PEG_2k_-LNP group. Similar fever levels were observed in the PEG_1k_-LNP and PEG_5k_-LNP groups (Figure 3B). In contrast, the PEG_1k_-LNP group exhibited a significant reduction in body weight 24 h postadministration compared with the PEG_2k_-LNP group (Figure 3C).

**Figure 3:**
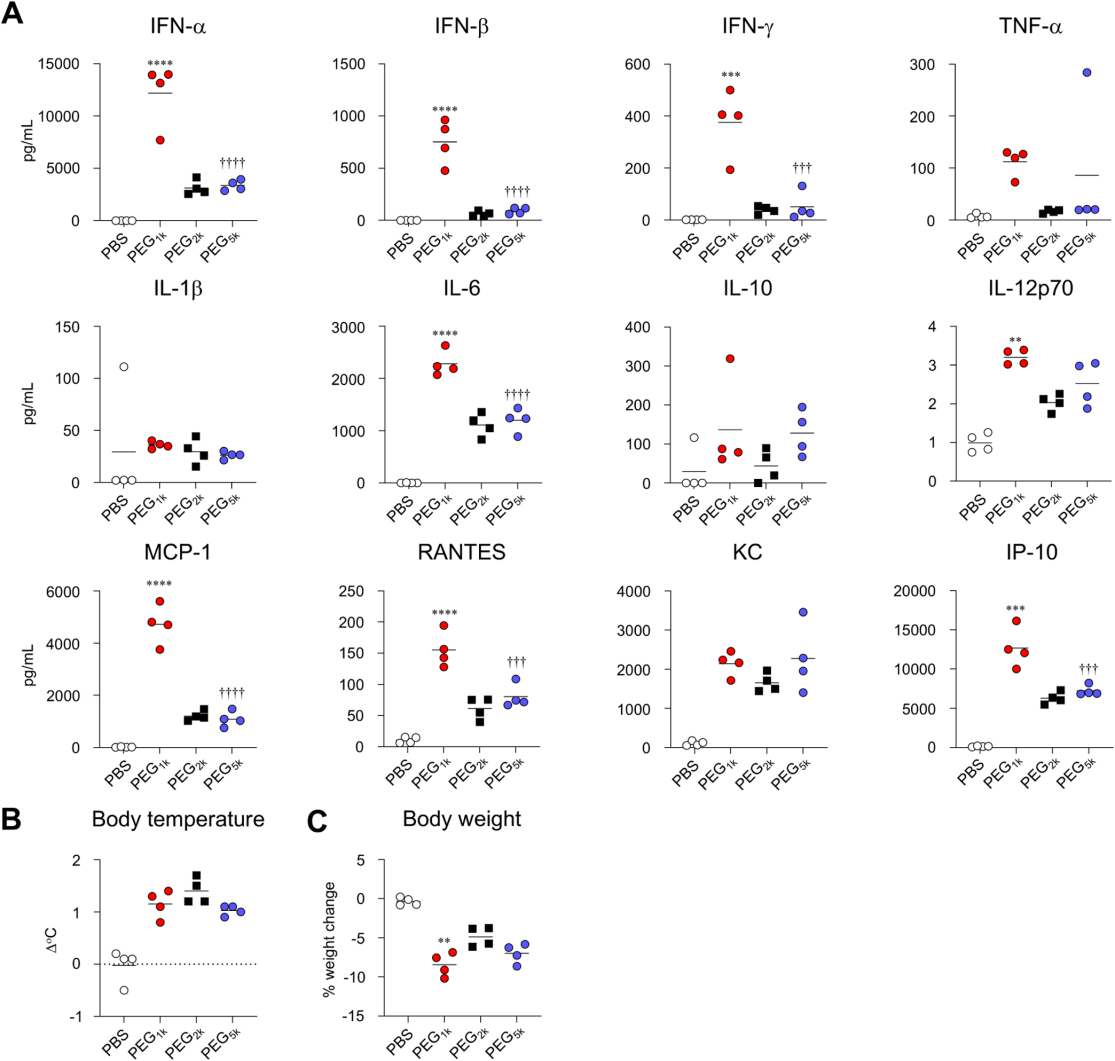
Effect of chain length of PEG on inflammatory cytokine production and adverse reactions induced by mRNA-LNPs. (A–C) Mice were intramuscularly administrated with S-LNPs. (A) Concentrations of IFN-α, IFN-β, IFN-γ, TNF-α, IL-1β, IL-6, IL-10, IL-12p70, MCP-1, RANTES, KC, and IP-10 in plasma were measured by multiplex assay at 6 h postadministration. (B) Changes in rectal temperature before and 6 h after administration were measured by a rectal temperature probe. (C) Changes in body weight before and 24 h after administration were measured. **P < 0.01, ***P < 0.001, ****P < 0.0001 vs PEG_2k_; ^†††^ *P* < 0.001, ^††††^ *P* < 0.0001 vs PEG_1k_ as determined by one-way ANOVA and Tukey’s multiple comparison test.

Together, changes in the PEG chain length altered the induction of antigen-specific CD8^+^ T cell responses and cytokine production but did not significantly alter antigen-specific antibody levels and fever.

### Effect of PEG-Lipid Molar Ratio

Next, we evaluated how varying the molar ratio of PEG-lipids affects immune responses and adverse reactions. The molar ratio of PEG_2k_-DMG in the control LNP was 1.5%. We formulated LNPs with PEG-lipid molar ratios ranging from 0% to 5.5% by altering the molar ratios of PEG_2k_-DMG and cholesterol (0%-LNP to 5.5%-LNP). Particle characteristics (Figure 4A) showed a PEG density-dependent decrease in particle size. At 6 h postadministration of FLuc-LNPs, the highest protein expression was observed in the 1.5%-LNP group in the TA, dLN, and liver, whereas in the spleen, the highest expression was observed in the 0.5%-LNP group (Figure 4B). In contrast, LNPs with a higher ratio of PEG (4.5%-LNP and 5.5%-LNP groups) showed decreased protein expression in all organs compared to the 1.5%-LNP group (Figure 4B). A similar trend was observed 24 h postadministration (Figure 4C). At both time points, LNPs with low PEG-lipid molar ratios, such as the 0%-LNP and 0.5%-LNP groups, showed lower protein expression levels in the TA, and liver than the 1.5%-LNP group (Figure 4B, C), indicating that reducing PEG-lipid molar ratio does not necessarily increase *in vivo* protein expression efficiency.

**Figure 4:**
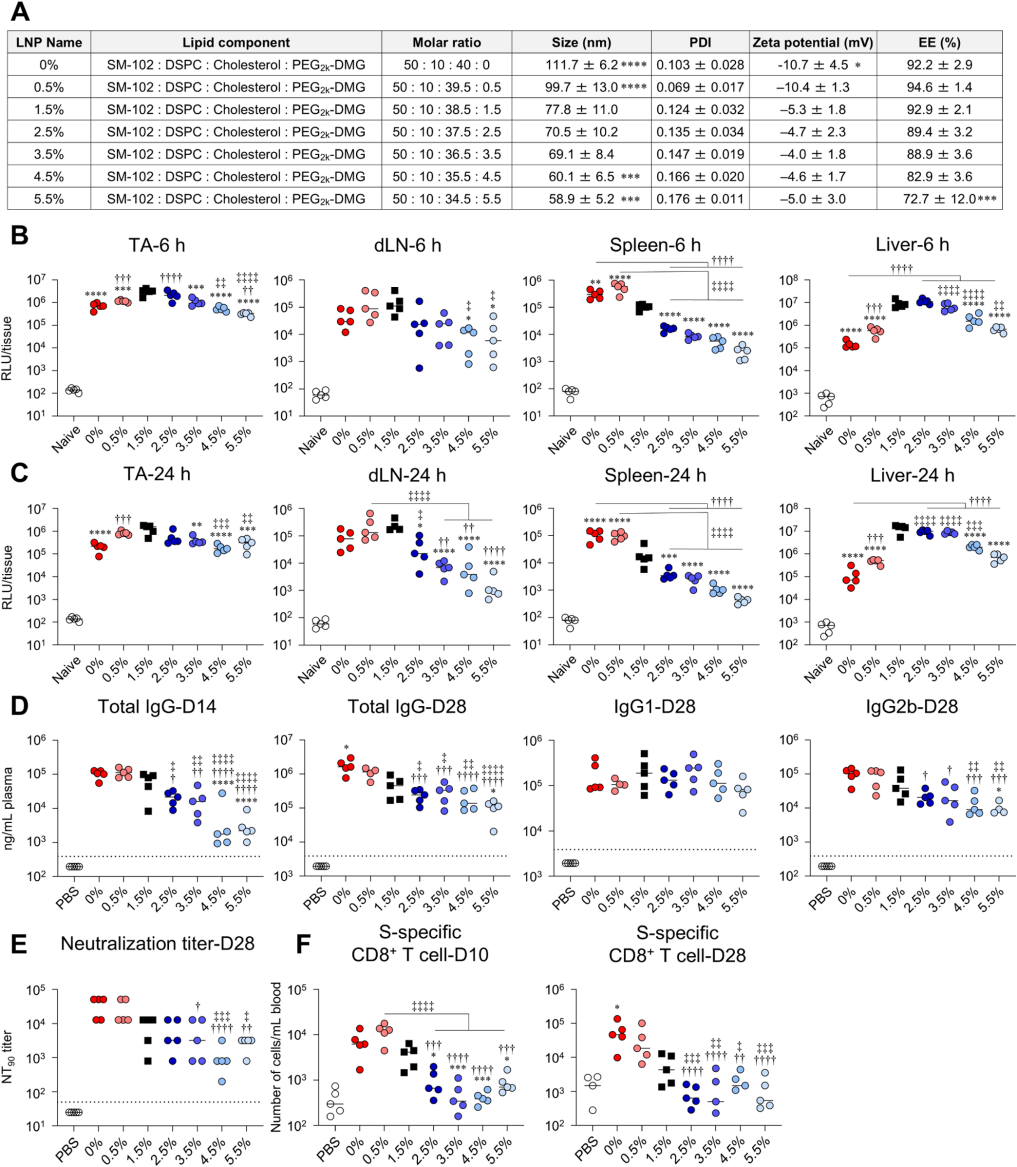
Effect of molar ratio of PEG-lipid on *in vivo* protein expression and immune responses induced by mRNA-LNPs. (A) Lipid composition and characteristics of LNPs with different PEG-lipid molar ratios. (B, C) Mice were intramuscularly administrated with FLuc-LNPs. FLuc activity in the homogenate of TA, dLN, spleen, and liver collected at (B) 6 h and (C) 24 h postadministration was measured as RLU. The results for the naive groups in (B) and (C) are identical. (D–F) Mice were vaccinated intramuscularly with S-LNPs. (D) S-specific IgG in the plasma on day 14 and S-specific IgG, IgG1, and IgG2b on day 28 were evaluated using ELISA. The dotted lines indicate the limit of detection. (E) Evaluation of neutralization activity of serum on day 28 against VSV-based pseudotyped viruses expressing the S protein of SARS-CoV-2 and determination of NT_90_ (F) S-specific CD8^+^ T cells in blood at days 10 and 28 were analyzed via flow cytometry. *P < 0.05, **P < 0.01, ***P < 0.001, ****P < 0.0001 vs 1.5%; ^†^ *P* < 0.05, ^††^ *P* < 0.01, ^†††^ *P* < 0.001, ^††††^ *P* < 0.0001 vs 0%; ^‡^ *P* < 0.05, ^‡‡^ *P* < 0.01, ^‡‡‡^ *P* < 0.001, ^‡‡‡‡^ *P* < 0.0001 vs 0.5% as determined by one-way ANOVA and Tukey’s multiple comparison test.

Following prime and boost vaccinations, the induction levels of S-specific IgG and IgG2b were dependent on the PEG molar ratio (Figure 4D). The groups receiving 0%-LNP exhibited significantly higher S-specific IgG production on day 28 than the 1.5%-LNP group, whereas those receiving 4.5%-LNP or 5.5%-LNP exhibited significantly less induction on days 14 and 28 (Figure 4D). Similarly, IgG2b levels in the 5.5%-LNP groups were lower than those in the 1.5%-LNP group, whereas IgG1 levels among the induced IgGs were comparable across all groups (Figure 4D). In correlation with S-specific IgG production, the NT_90_ and NT_99_ levels were significantly lower in the 3.5-LNP, 4.5%-LNP, and 5.5%-LNP groups than they were in the 0%-LNP and 0.5%-LNP groups (Figures 4E and S2B). In addition, the induction of S-specific CD8^+^ T cells was dependent on the PEG-lipid ratio (Figure 4F). The number of S-specific CD8^+^ T cells in the blood after prime and boost vaccination was significantly higher in the 0%-LNP group than in the 1.5%-LNP group; however, it was significantly lower in the group treated with a PEG ratio of 2.5% or higher (Figure 4F).

Quantification of systemic cytokine production following LNP administration revealed that compared with the 1.5%-LNP group, the 0.5%-LNP group exhibited significantly higher levels of type I IFN, IFN-γ, TNF-α, and various chemokines, including MCP-1, RANTES, and IP-10, whereas the 0%-LNP group only exhibited higher TNF-α and MCP-1 levels (Figure 5A). In contrast, the groups treated with LNPs with a PEG ratio of 4.5% or higher tended to produce lower levels of all cytokines and chemokines compared to the 1.5%-LNP group, showing significantly reduced production of type I IFN, RANTES, and IP-10 (Figure 5A). No significant differences in cytokine levels were observed between the 2.5%-LNP and 1.5%-LNP groups (Figure 5A). In the groups treated with 0%-LNP, 0.5%-LNP, and 1.5%-LNP, an increase in rectal temperature ranging from 0.5 to 1 °C was observed at 6 h postadministration (Figure 5B). However, the groups administered LNPs with a PEG ratio of 2.5% or higher exhibited a significant reduction in fever compared with the 1.5%-LNP group (Figure 5B). The percentage change in body weight was similar in all groups (Figure 5C).

**Figure 5:**
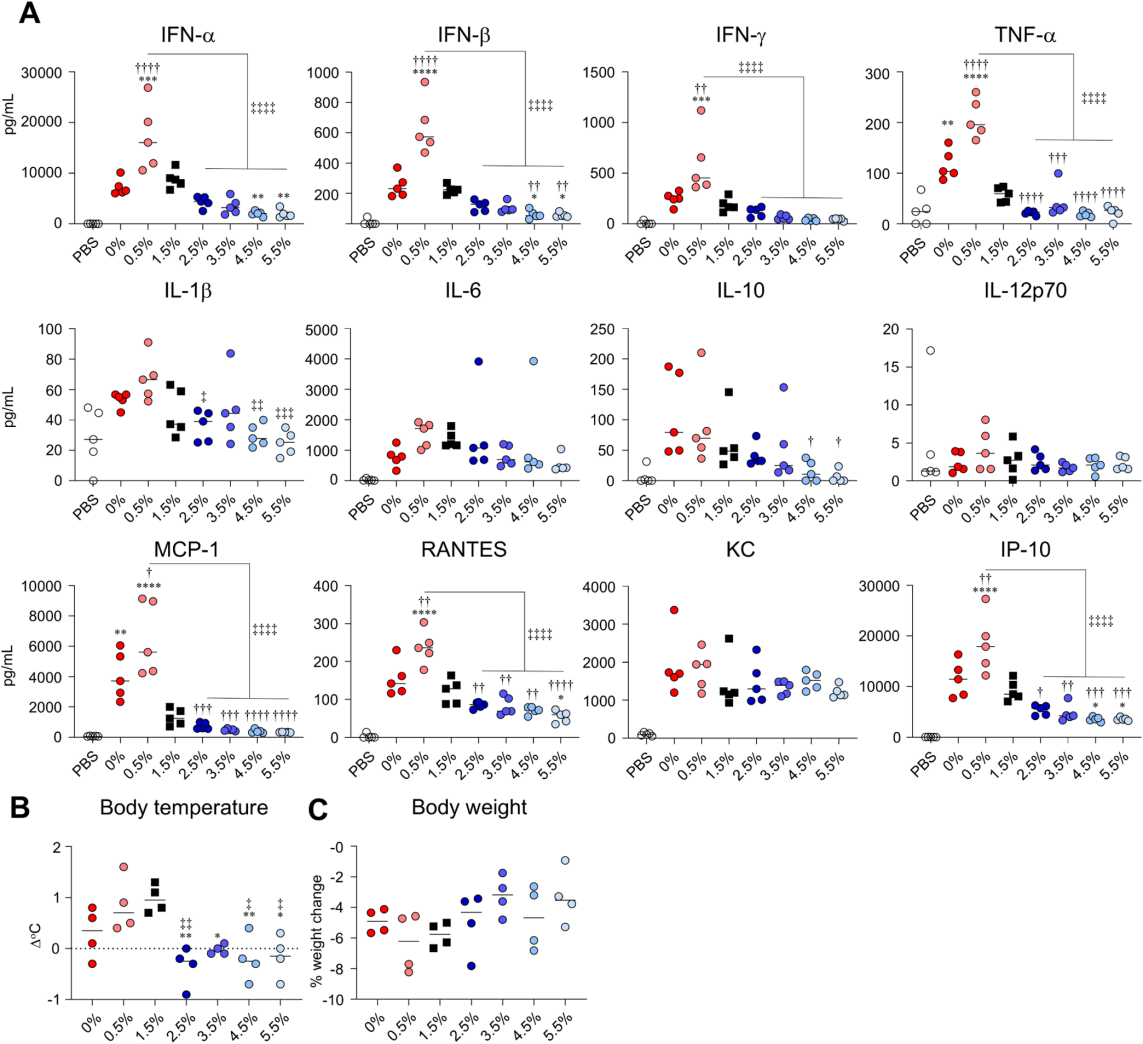
Effect of molar ratio of PEG-lipid on inflammatory cytokine production and adverse reactions induced by mRNA-LNPs. (A–C) Mice were intramuscularly administrated with S-LNPs. (A) Concentrations of IFN-α, IFN-β, IFN-γ, TNF-α, IL-1β, IL-6, IL-10, IL-12p70, MCP-1, RANTES, KC, and IP-10 in plasma were measured by multiplex assay at 6 h postadministration. (B) Changes in rectal temperature before and 6 h after administration were measured by a rectal temperature probe. (C) Changes in body weight before and 24 h after administration were measured. *P < 0.05, **P < 0.01, ***P < 0.001, ****P < 0.0001 vs 1.5%; ^†^ *P* < 0.05, ^††^ *P* < 0.01, ^†††^ *P* < 0.001, ^††††^ *P* < 0.0001 vs 0%; ^‡^ *P* < 0.05, ^‡‡^ *P* < 0.01, ^‡‡‡^ *P* < 0.001, ^‡‡‡‡^ *P* < 0.0001 vs 0.5% as determined by one-way ANOVA and Tukey’s multiple comparison test.

Together, PEG modification rates were negatively correlated with immune induction but did not correlate with protein expression, cytokine production, or fever.

### Effect of Replacement of Cholesterol with Naturally Occurring Cholesterol Analogs

We focused on cholesterol and evaluated the effects of cholesterol analog replacement on protein expression and immune responses. Three cholesterol derivatives were selected: stigmastanol, β-sitosterol, and campesterol. These derivatives introduced methyl or ethyl groups at C-24 of cholesterol (Figure 6A-i). LNPs substituted with these derivatives (Stig-LNP, Sito-LNP, Camp-LNP) exhibited similar particle characteristics but significantly enhanced transfection efficiency *in vitro* compared with cholesterol-containing LNP (Chol-LNP) as previously reported^36^ (Figure 6A-ii,iii). At 6 h postadministration of FLuc-LNPs, similar expression levels were observed in the TA in all groups (Figure 6B). Lower expression levels were observed in the spleen and liver in the Stig-LNP and Sito-LNP groups than in the Chol-LNP group (Figure 6B). Similar trends were observed at 24 h, where both Stig-LNP and Sito-LNP groups showed significantly lower expression in the spleen and liver compared with the Chol-LNP group (Figure 6C). These results showed that although the exact mechanisms remain unclear, Stig-LNPs, Sito-LNPs, and Camp-LNPs did not enhance *in vivo* protein expression compared with Chol-LNPs, unlike the improvement *in vitro* transfection efficiency.

**Figure 6:**
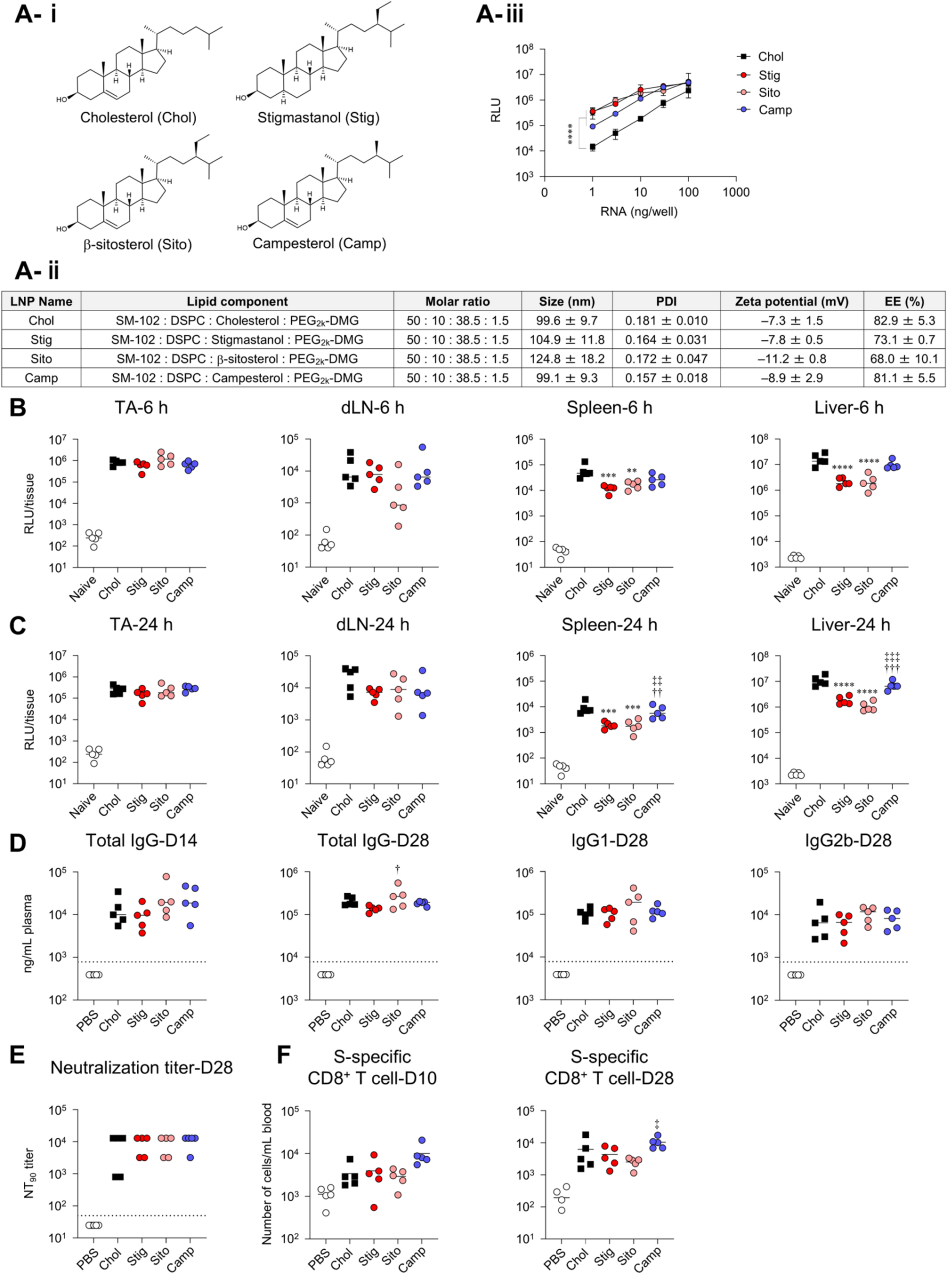
Effect of replacement of cholesterol with naturally occurring cholesterol analogs on *in vivo* protein expression and immune responses induced by mRNA-LNPs. (A-i) Chemical structures of cholesterol, stigmastanol, β-sitosterol, and campesterol. (A-ii) Lipid composition and characteristics of LNPs replaced cholesterols with naturally occurring cholesterol analogs. (A-iii) *in vitro* protein expression analysis. FLuc-LNPs were transfected into A549 cells, and the FLuc activity in the cell lysate was measured as RLU. (B, C) Mice were intramuscularly administrated with FLuc-LNPs. FLuc activity in the homogenate of TA, dLN, spleen, and liver collected at (B) 6 h and (C) 24 h postadministration was measured as RLU. The results for the naive groups in (B) and (C) are identical. (D–F) Mice were vaccinated intramuscularly with S-LNPs. (D) S-specific IgG in the plasma on day 14 and S-specific IgG, IgG1, and IgG2b on day 28 were evaluated using ELISA. The dotted lines indicate the limit of detection. (E) Evaluation of neutralization activity of serum on day 28 against VSV-based pseudotyped viruses expressing the S protein of SARS-CoV-2 and determination of NT_90_. (F) S-specific CD8^+^ T cells in blood at days 10 and 28 were analyzed via flow cytometry. *P < 0.05, **P < 0.01, ***P < 0.001, ****P < 0.0001 vs Chol; ^†^ *P* < 0.05, ^††^ *P* < 0.01, ^†††^ *P* < 0.001 vs Stig; ^‡^ *P* < 0.05, ^‡‡^ *P* < 0.01, ^‡‡‡^ *P* < 0.001 vs Sito as determined by one-way ANOVA and Tukey’s multiple comparison test.

Next, we evaluated immune responses. Following prime and boost vaccinations, S-specific IgG levels, as well as IgG1 and IgG2b subclasses, were comparable between Chol-LNP and other LNPs (Figure 6D). Correlating with this result, similar NT_90_ and NT_99_ titers were shown in all groups (Figures 6E and S2C). Similar levels of S-specific CD8^+^ T cell responses were induced between Chol-LNP group and other LNP groups after both vaccinations (Figure 6F).

Systemic cytokine responses revealed that the Stig-LNP and Sito-LNP groups produced lower levels of most cytokines and chemokines compared with those in the Chol-LNP-treated group, with significant reductions observed in the production of IFN-α, IFN-γ, TNF-α, MCP-1, RANTES, and IP-10, whereas the Camp-LNP group showed a significant reduction only in TNF-α (Figure 7A). Furthermore, the Stig-LNP group demonstrated a significant reduction in fever compared with the Chol-LNP group at 6 h postadministration, whereas body weight changes were similar in all groups (Figure 7B, C).

**Figure 7:**
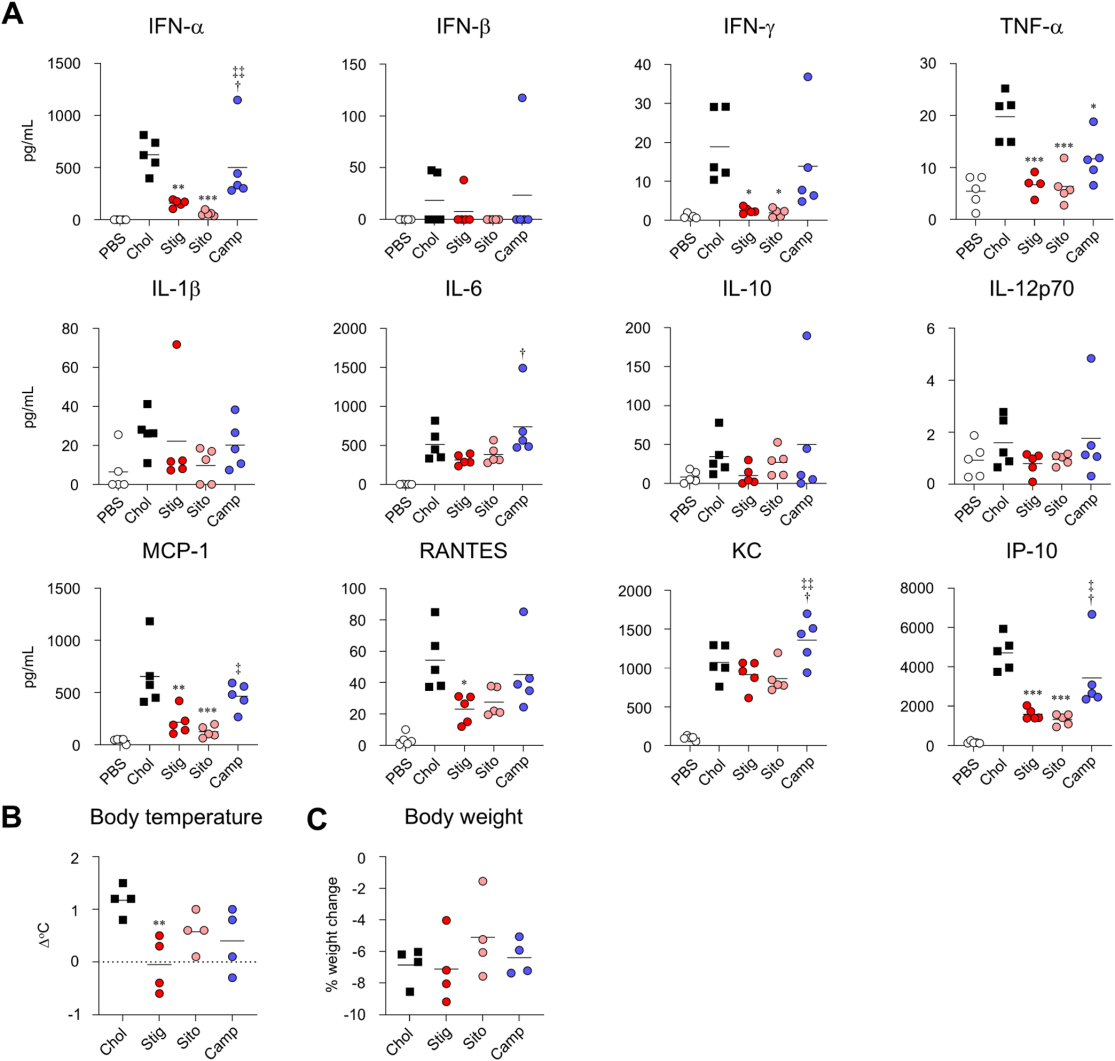
Effect of replacement of cholesterol with naturally occurring cholesterol analogs on inflammatory cytokine production and adverse reactions induced by mRNA-LNPs. (A–C) Mice were intramuscularly administrated with S-LNPs. (A) Concentrations of IFN-α, IFN-β, IFN-γ, TNF-α, IL-1β, IL-6, IL-10, IL-12p70, MCP-1, RANTES, KC, and IP-10 in plasma were measured by multiplex assay at 6 h after administration. (B) Changes in rectal temperature before and 6 h after administration were measured by a rectal temperature probe. (C) Changes in body weight before and 24 h after administration were measured. *P < 0.05, **P < 0.01, ***P < 0.001 vs Chol; ^†^ *P* < 0.05 vs Stig; ^‡^ *P* < 0.05, ^‡‡^ *P* < 0.01 vs Sito as determined by one-way ANOVA and Tukey’s multiple comparison test.

Taken together, both the Stig-LNP and Sito-LNP groups exhibited reduced cytokine production, with the Stig-LNP group also lowering fever while inducing similar levels of immune response as the control LNP.

### Effect of Replacement of Phospholipids

We evaluated the effects of phospholipids on protein expression and immune responses. Currently approved mRNA-LNP vaccines utilize DSPC, which contains stearic acid, a saturated fatty acid, as its acyl group and phosphatidylcholine as its phosphate group (Figure 8A-i). In this study, we prepared and evaluated LNPs in which DSPC was completely replaced by dioleoylphosphatidylcholine (DOPC) containing oleic acid, an unsaturated fatty acid, as the acyl group, and dioleoylphosphatidylethanolamine (DOPE) containing oleic acid as the acyl group and phosphatidylethanolamine as the phosphate group (Figure 8A-i). Compared with the control formulation containing DSPC (DSPC-LNP), LNPs containing DOPC or DOPE (DOPC-LNP, DOPE-LNP) exhibited similar encapsulation efficiency (Figure 8A-ii). However, increased particle size was observed in DOPE-LNP, and both DOPC-LNP and DOPE-LNP showed elevated zeta potential (Figure 8A-ii). At 6 h postadministration of FLuc-LNPs, similar levels of FLuc expression were observed in the TA across all groups. However, in the DOPC-LNP group, decreased expression was observed in the dLN, spleen, and liver (Figure 8B). In the DOPE-LNP group, the protein expression was reduced exclusively in the dLN, whereas in other organs, it remained comparable to that of the DSPC-LNP group (Figure 8B). At 24 h postadministration, the DOPC-LNP group exhibited lower protein expression in the TA, spleen, and liver. The DOPE-LNP group also demonstrated lower expression levels in the spleen, indicating a decrease in expression in distant organs (Figure 8C).

**Figure 8:**
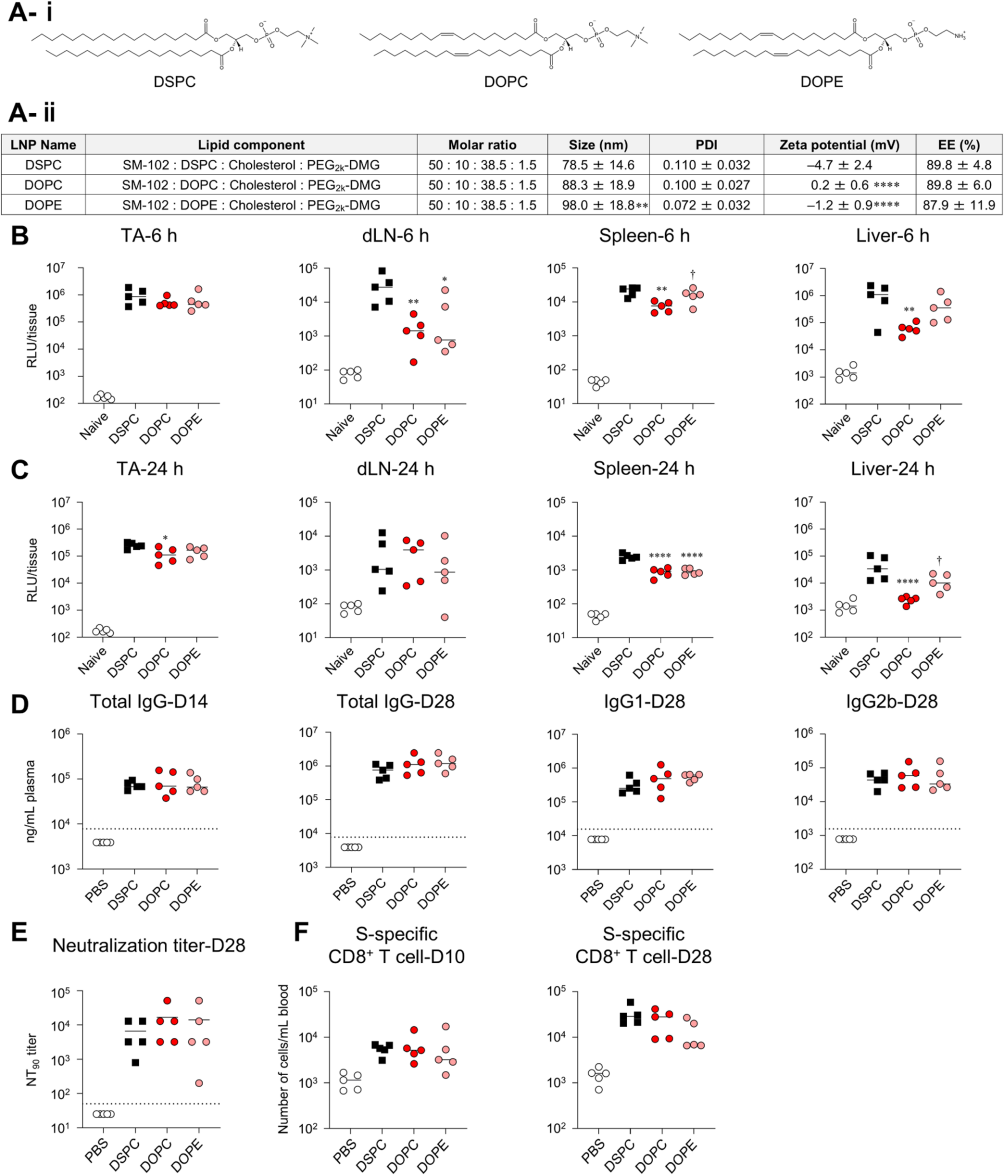
Effect of replacement of phospholipid on *in vivo* protein expression and immune responses induced by mRNA-LNPs. (A-i) Chemical structures of DSPC, DOPC, and DOPE. (A-ii) The lipid composition and characteristics of LNPs replaced DSPC with DOPC and DOPE. (B, C) Mice were intramuscularly administrated with FLuc-LNPs. FLuc activity in the homogenate of TA, dLN, spleen, and liver collected at (B) 6 h and (C) 24 h postadministration was measured as RLU. The results for the naive groups in (B) and (C) are identical. (D–F) Mice were vaccinated intramuscularly with S-LNPs. (D) S-specific IgG in the plasma on day 14 and S-specific IgG, IgG1, and IgG2b on day 28 were evaluated using ELISA. The dotted lines indicate the limit of detection. (E) Evaluation of neutralization activity of serum on day 28 against VSV-based pseudotyped viruses expressing the S protein of SARS-CoV-2 and determination of NT_90_. (F) S-specific CD8^+^ T cells in blood at days 10 and 28 were analyzed via flow cytometry. *P < 0.05, **P < 0.01, ****P < 0.0001 vs Chol; ^†^ *P* < 0.05 vs DOPC as determined by one-way ANOVA and Tukey’s multiple comparison test.

The levels of S-specific IgG and its IgG1 and IgG2b subclasses were comparable across all groups after both prime and boost vaccinations (Figure 8D). Correlating with this result, similar NT_90_ and NT_99_ titers were shown in all groups (Figures 8E and S2D). Furthermore, the number of S-specific CD8^+^ T cells was similar to that in the DSPC-LNP group, whereas the DOPE-LNP group exhibited a significantly lower induction of S-specific CD8^+^ T cells after boost vaccination (Figure 8F).

In addition, the DOPC-LNP and DOPE-LNP groups exhibited significantly lower levels of IFN-α, IFN-γ, TNF-α, MCP-1, IL-1β, IL-12p70, MCP-1, and IP-10 (Figure 9A). The DOPC-LNP group showed significantly lower levels of RANTES than did the DSPC-LNP group (Figure 9A). Additionally, at 3 h postadministration of DSPC-LNP and DOPC-LNP, levels of some cytokines and chemokines, especially KC, were significantly lower in the DOPC-LNP group compared with that in the DSPC-LNP groups (Figure S3). These results suggest that some cytokines and chemokines reach their peak production earlier than 6 h postadministration and exhibit differences in concentration at earlier time points similar to those observed for other cytokines. Both the DOPC-LNP and DOPE-LNP groups exhibited a significant reduction in fever compared with the DSPC-LNP group (Figure 9B). Weight changes after vaccination were similar in all groups (Figure 9C).

**Figure 9:**
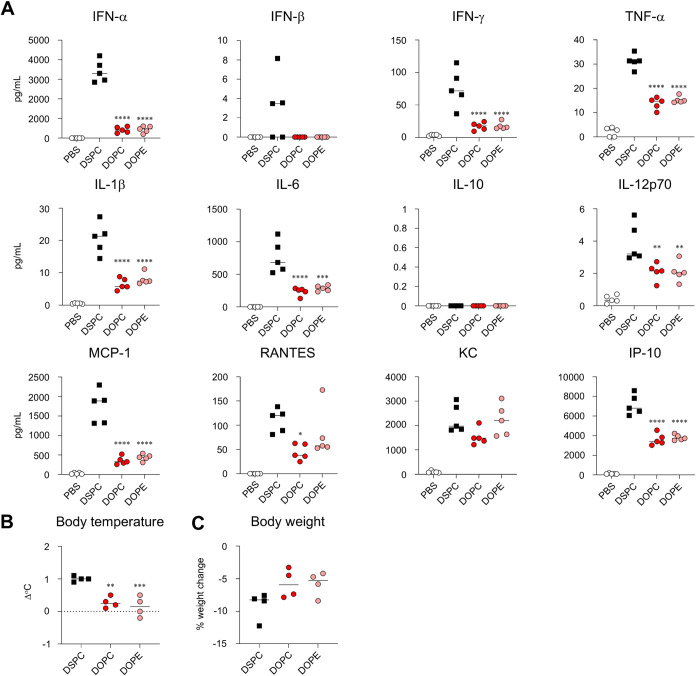
Effect of replacement of phospholipid on inflammatory cytokine production and adverse reactions induced by mRNA-LNPs. (A–C) Mice were intramuscularly administrated with S-LNPs. (A) Concentrations of IFN-α, IFN-β, IFN-γ, TNF-α, IL-1β, IL-6, IL-10, IL-12p70, MCP-1, RANTES, KC, and IP-10 in plasma were measured by multiplex assay at 6 h postadministration. (B) Changes in rectal temperature before and 6 h after administration were measured by a rectal temperature probe. (C) Changes in body weight before and 24 h after administration were measured. *P < 0.05, **P < 0.01, ***P < 0.001, ****P < 0.0001 vs Chol as determined by one-way ANOVA and Tukey’s multiple comparison test.

These results indicate that phospholipid-replaced LNPs, particularly DOPC-LNPs, induce an immune response comparable to that of the control LNP while reducing inflammatory and adverse reactions.

### Temporal Changes in S-Specific IgG Production and Adverse Reactions

To assess comprehensive immune responses and adverse reactions, we monitored plasma S-specific IgG levels and adverse reactions over an extended period. Representative formulations (control-LNP, PEG_1k_-LNP, PEG0%-LNP, PEG0.5%-LNP, DOPC-LNP, and Stig-LNP) were selected for evaluation (Figure 10A). For the plasma S-specific IgG levels, no significant difference in antibody levels was observed among all the groups up to day 105, indicating that none of the tested formulations exhibited reduced antibody durability (Figure 10B). For the evaluation of adverse reactions, mice were vaccinated with LNPs twice at a 3-week interval (20 μg mRNA/mouse). The results showed that blood cytokine levels peaked at 6–24 h postprime and postboost administration (Figure 11A, B). However, by days 7, 14, 20, 28, and 35, all groups returned to levels comparable to the PBS group, indicating that cytokine production was not sustained over the subsequent weeks (Figure 11A). Additionally, similar to the cytokine response, fever and body weight reduction peaked at 6 and 24 h following both prime and boost administration, respectively, but returned to levels comparable to those of the PBS group within 7 days postprime and postboost administration (Figure 12A,B). No prolonged weight loss was observed in any of the groups (Figure 12C).

**Figure 10:**
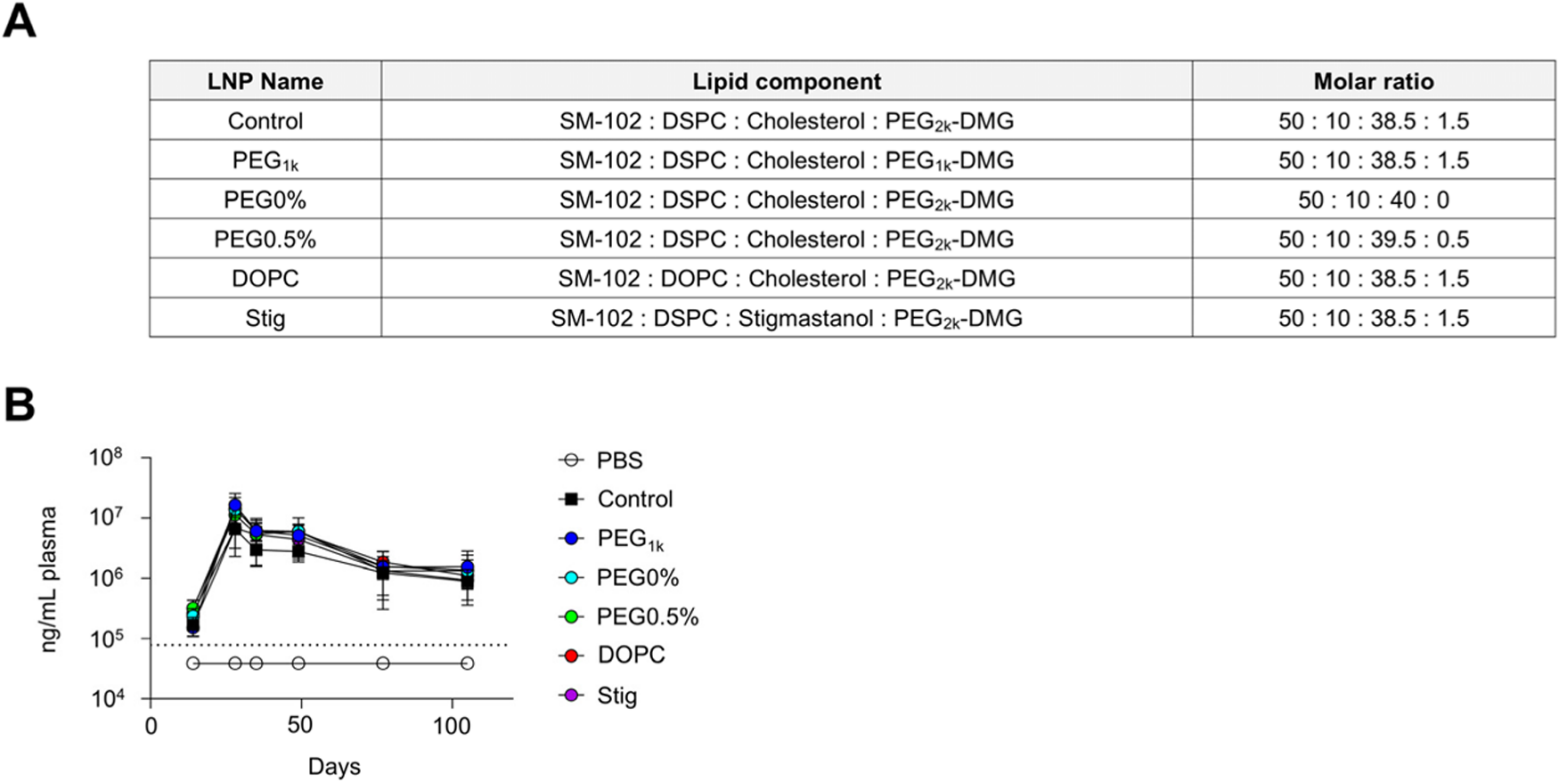
Temporal changes in the S-specific IgG response induced by mRNA-LNPs. (A) Lipid composition of LNPs. (B) Mice were vaccinated intramuscularly with S-LNPs. Plasma S-specific IgG levels on days 14, 28, 35, 49, 77, and 105 were evaluated using ELISA. Dotted lines indicate the limit of detection.

**Figure 11:**
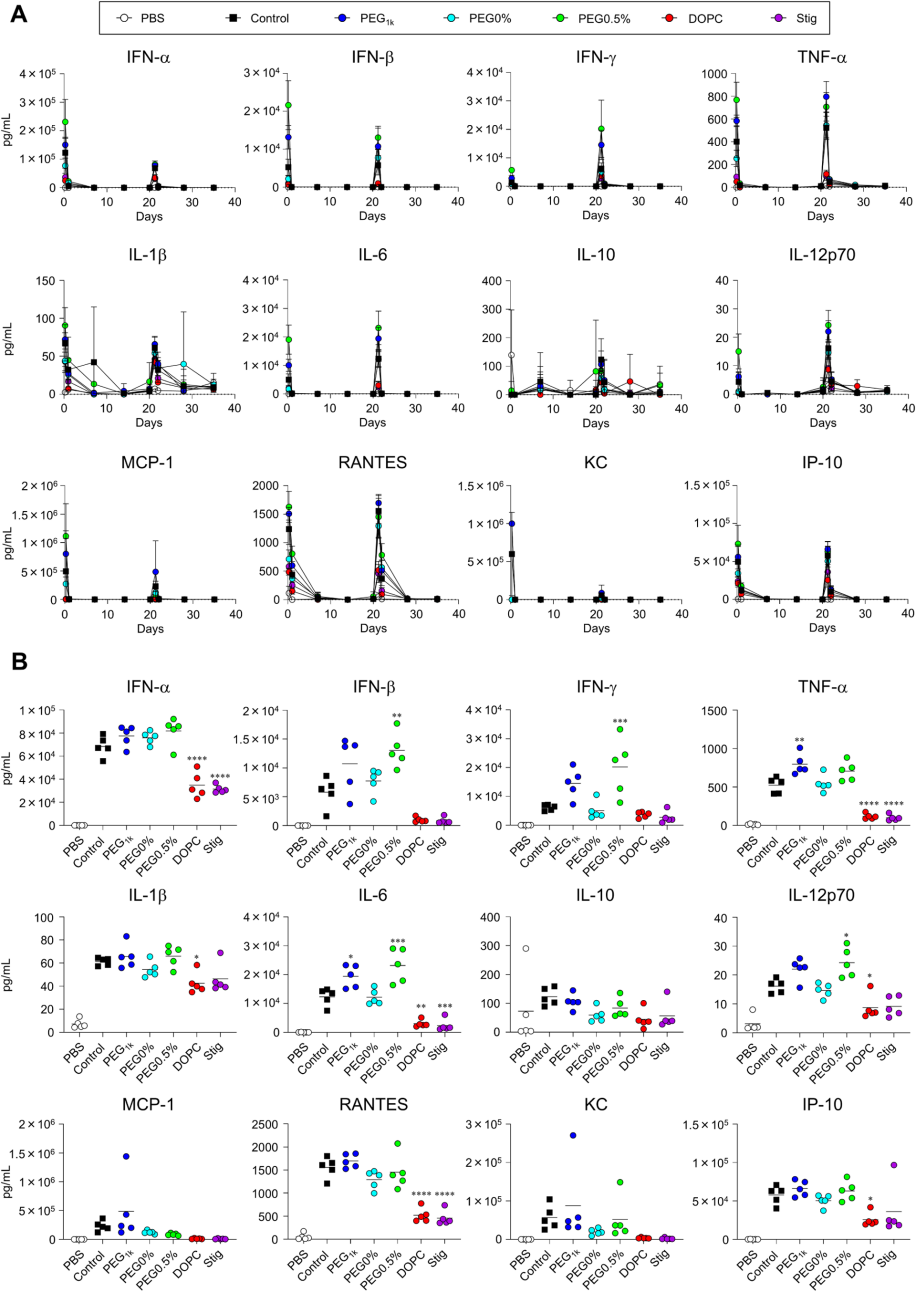
Temporal changes in inflammatory cytokine production induced by mRNA-LNPs. Mice were intramuscularly administrated with S-LNPs twice at 3-week intervals (20 μg mRNA/mouse). (A) Concentrations of IFN-α, IFN-β, IFN-γ, TNF-α, IL-1β, IL-6, IL-10, IL-12p70, MCP-1, RANTES, KC, and IP-10 in the plasma at 6 and 24 h, 7, 14, and 21 days after prime administration, and at 6 and 24 h and 7 and 14 days after boost administration. (B) Cytokine levels at 6 h postboost administration, extracted from the corresponding values shown in (A). *P < 0.05, **P < 0.01, ***P < 0.001, ****P < 0.0001 vs control as determined by one-way ANOVA and Tukey’s multiple comparison test.

**Figure 12:**
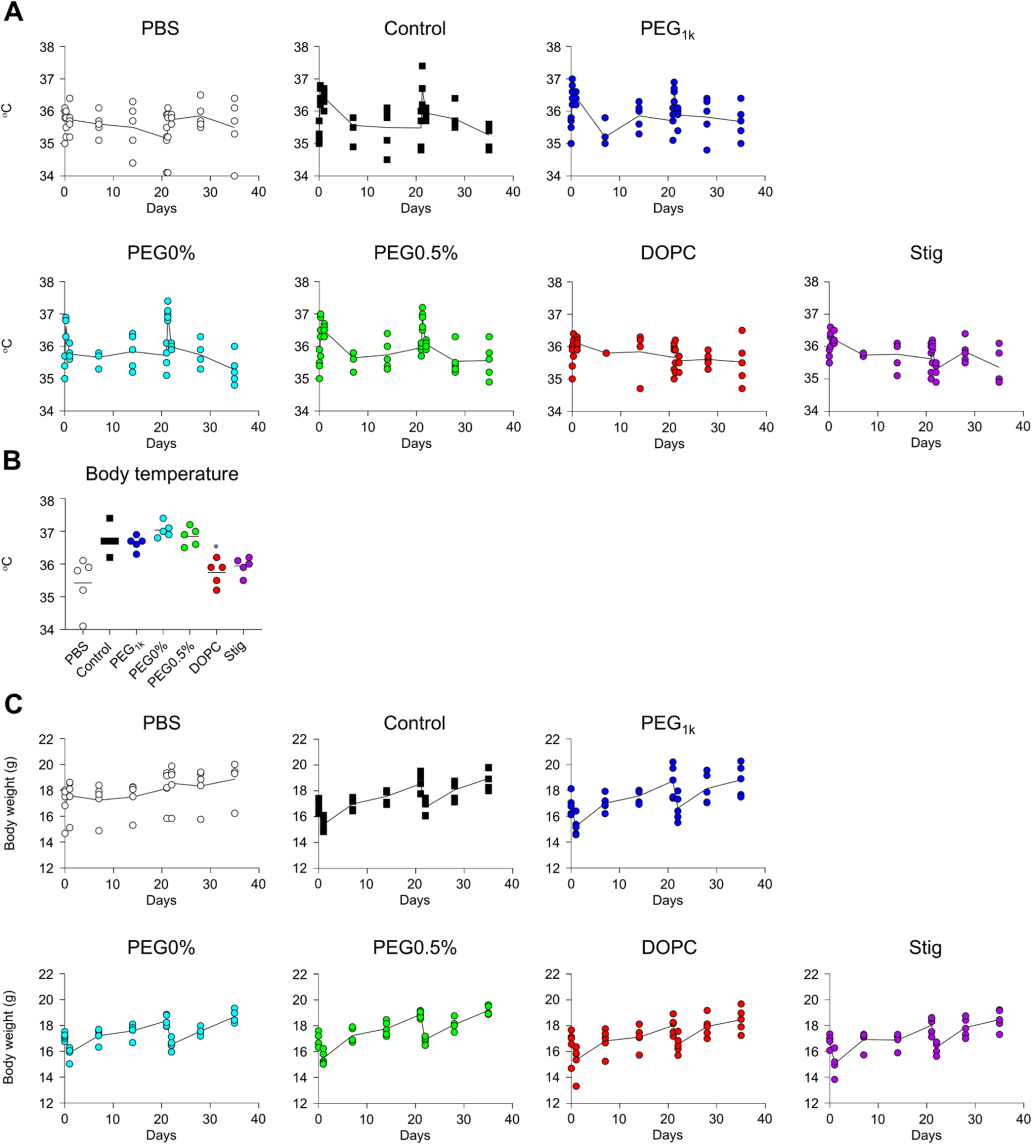
Temporal changes in fever and body weight induced by mRNA-LNPs. Mice were intramuscularly administrated with S-LNPs twice at 3-week intervals (20 μg mRNA/mouse). (A) Rectal temperature measured at 6 and 24 h and 7, 14, and 21 days after prime administration and at 6 and 24 h and 7 and 14 days after boost administration by a rectal temperature probe. (B) Rectal temperature at 6 h postboost administration, extracted from the corresponding values shown in (A). (C) Body weight at 24 h and 7, 14, and 21 days after prime administration and at 24 h and 7 and 14 days after boost administration *P < 0.05 vs control as determined by one-way ANOVA and Tukey’s multiple comparison test.

These results indicate that all the LNP formulations tested in this experiment elicited comparable S-specific IgG kinetics and did not induce adverse reactions such as fever or body weight loss over an extended period.

### Stability of LNPs

Given the importance of LNP stability as a critical factor for the clinical application of optimized mRNA-LNPs, we evaluated the stability of the LNP formulations identified in this study. A selection of representative formulations (control-LNP, PEG_1k_-LNP, PEG0%-LNP, PEG0.5%-LNP, DOPC-LNP, and Stig-LNP) was used to evaluate stability (Figure S4A). The formulations were stored at −20, 4, and 25 °C for 21 days, and particle size, proportion of encapsulated mRNA, and transfection efficiency into HEK293 cells were assessed at 7-day intervals. Although sucrose, a cryoprotectant, is often added during the storage of LNPs, PBS was used as the solvent in this study in order to evaluate the intrinsic stability differences among individual LNPs. The results showed that when stored at −20 °C, all formulations exhibited an increase in particle size at all time points (Figure S4B). Notably, PEG0%-LNP demonstrated a clear increase in particle size and marked reductions in the proportion of encapsulated mRNA and *in vitro* transfection efficiency after storage (Figure S4B). These findings indicate that PEG0%-LNPs have poor stability at – 20 °C or are susceptible to instability during freeze–thaw cycles. At 4 °C, Stig-LNP exhibited an increase in particle size as well as a decrease in proportion of encapsulated mRNA on day 7 (Figure S4C). For the PEG_1k_-LNP, PEG0%-LNP, and PEG0.5%-LNP, significant decreases in *in vitro* transfection efficiency were observed throughout the study period (Figure S4C). When stored at 25 °C, all formulations except for Stig-LNP exhibited a significant decrease in transfection efficiency over the study period (Figure S4D). In contrast, all formulations maintained stable particle size and proportion of encapsulated mRNA throughout the same period (Figure S4D).

These results indicate that some of the LNP formulations evaluated in this study exhibit stability issues when frozen or stored at 4 or 25 °C.

### Effect of Combined Modification of PEG-Lipids, Phospholipids, and Cholesterol

Building on the above results, we next designed LNP formulations in various combinations by varying the PEG-lipid molar ratio (from 1.5% to 0% or 0.5%), replacing DSPC with DOPC, and replacing cholesterol with stigmastanol, aiming to enhance immune responses while reducing adverse reactions. The physicochemical properties of the formulated LNPs are presented in Figure 13A. The DOPC substitution combined with PEG0.5% and the stigmastanol substitution combined with PEG0.5% resulted in increased particle size (∼200 nm), whereas the LNPs were successfully prepared. However, we were unable to formulate LNPs combining PEG0% and DOPC (DOPC-PEG0%), PEG0% and stigmastanol (Stig-PEG0%), and DOPC and stigmastanol (DOPC-Stig-PEG0.5% and DOPC-Stig-PEG0%). Furthermore, we also prepared LNPs incorporating DOPC and stigmastanol substitutions combined with PEG1.5%; however, these LNPs exhibited excessive aggregation (∼1 μm in size) and were therefore excluded from subsequent experiments. Based on the above results, further evaluation of immune responses and adverse reactions were conducted on the two final LNP formulations that were successfully prepared: one comprising DOPC and PEG0.5% and the other comprising stigmastanol and PEG0.5%.

**Figure 13:**
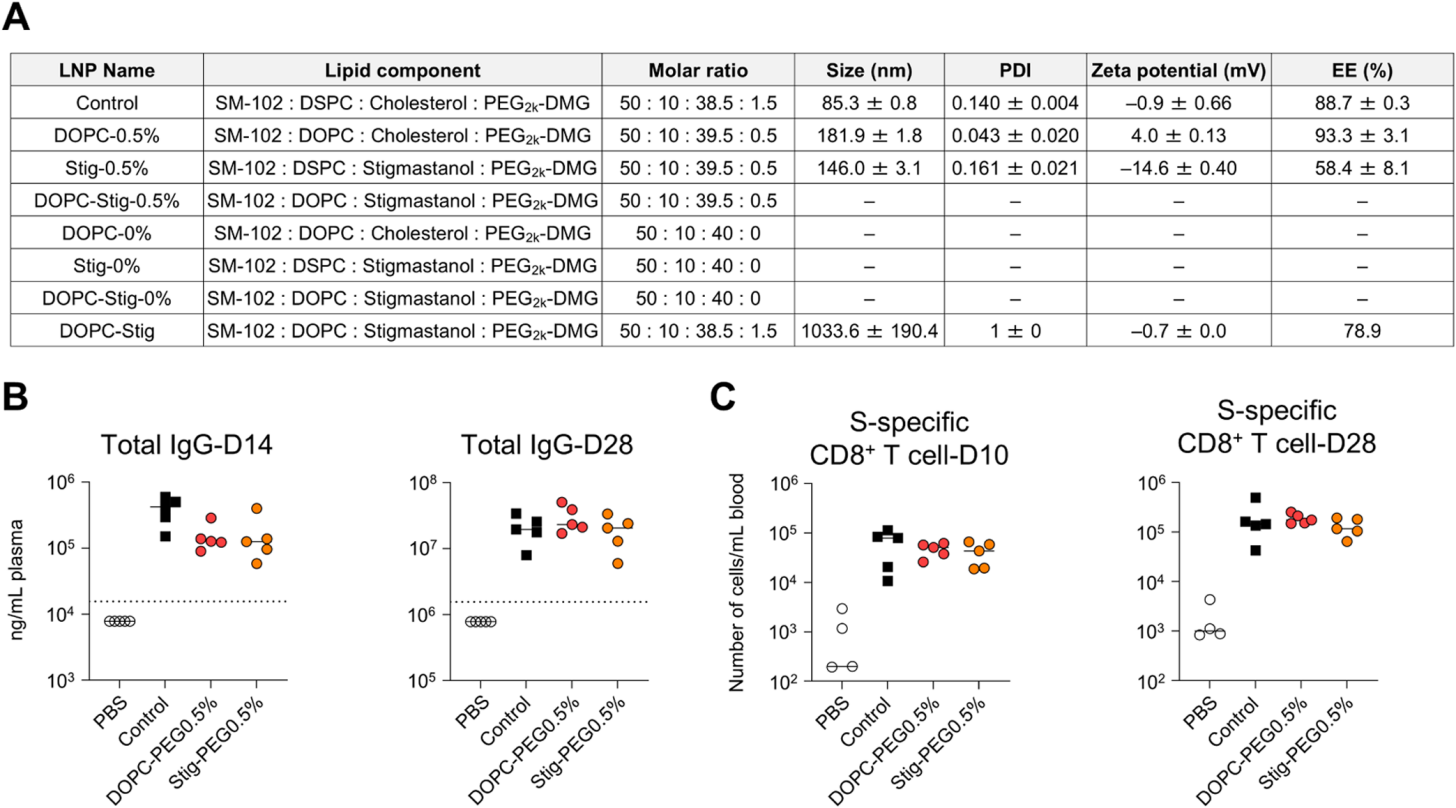
Effect of combined modification of PEG-lipids, phospholipids, and cholesterol on immune responses induced by mRNA-LNPs. (A) Lipid composition and characteristics of LNPs. ”–” indicates that LNPs could not be successfully formulated. (B, C) Mice were vaccinated intramuscularly with S-LNPs. (B) S-specific IgG levels in the plasma on days 14 and 28, evaluated using ELISA. Dotted lines indicate the limit of detection. (C) S-specific CD8^+^ T cells in the blood at days 10 and 28 were analyzed via flow cytometry.

Following prime and boost vaccinations, both the DOPC-PEG0.5% and Stig-PEG0.5% groups induced comparable levels of S-specific IgG to the control LNP group (DSPC-Chol-PEG1.5%) on days 14 and 28 (Figure 13B). Additionally, the numbers of S-specific CD8^+^ T cells in the blood on days 10 and 28 were also similar between these groups and the control LNP group (Figure 13C). In addition, quantification of systemic cytokine production following LNP administration revealed that both the DOPC-PEG0.5% and Stig-PEG0.5% groups showed significantly lower levels of TNF-α, IL-6, MCP-1, and RANTES than did the control LNP group at 6 h postprime administration (Figure 14A). The Stig-PEG0.5% group showed significantly lower levels of type I IFN, IL-10, IL-12p70, KC, and IP-10 than did the control LNP group (Figure 14A). Furthermore, the Stig-PEG0.5% group exhibited a significant reduction in fever compared with that of the control LNP group (Figure 14B). Weight changes after vaccination were similar in all groups (Figure 14C). These findings suggest that LNPs optimized for PEG-lipids and phospholipids, or PEG-lipids and cholesterol can effectively reduce adverse reaction. However, they do not enhance immune responses.

**Figure 14:**
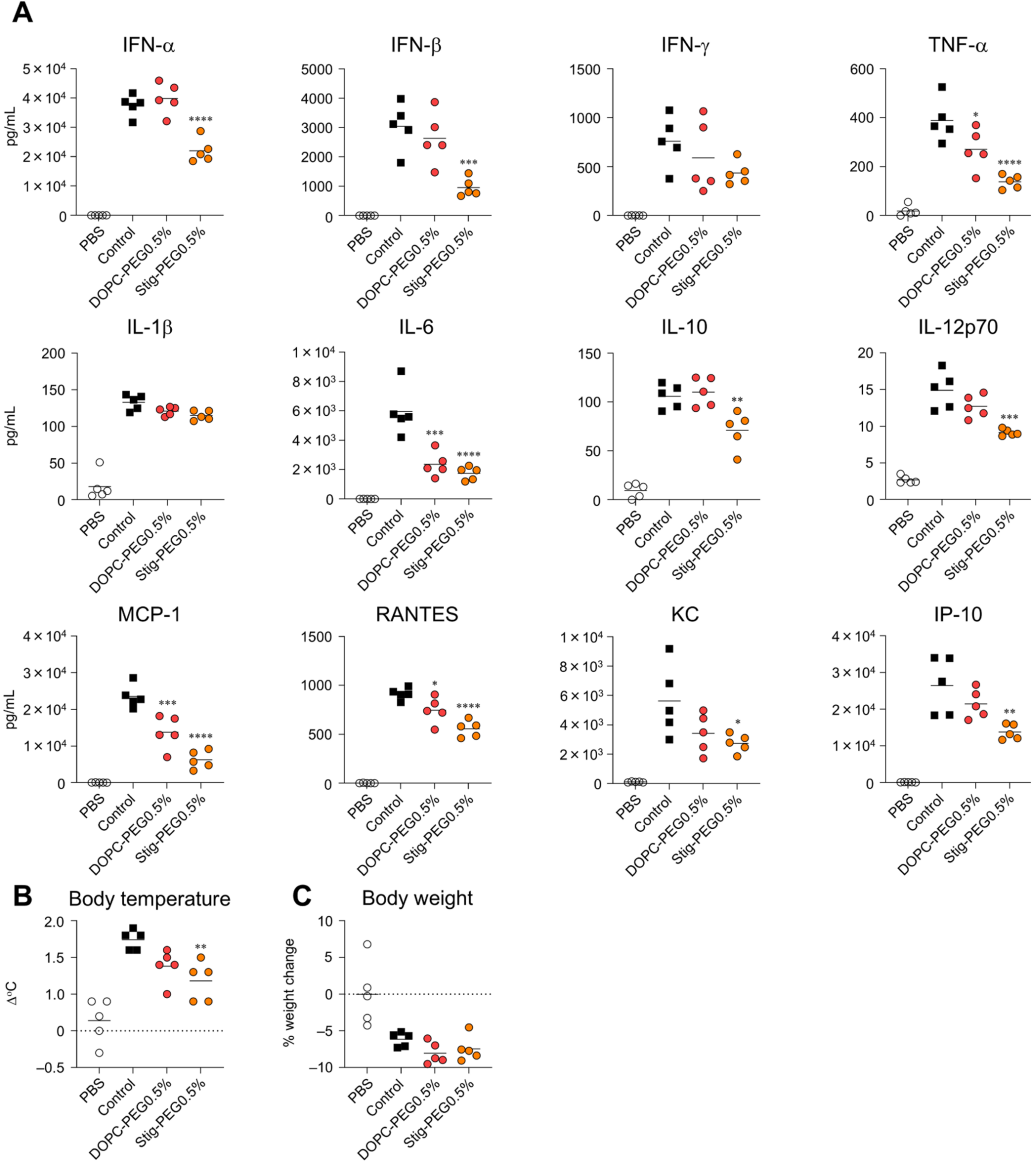
Effect of combined modification of PEG-lipids, phospholipids, and cholesterol on inflammatory cytokine production and adverse reactions induced by mRNA-LNPs. (A–C) Mice were intramuscularly administrated with S-LNPs. (A) Concentrations of IFN-α, IFN-β, IFN-γ, TNF-α, IL-1β, IL-6, IL-10, IL-12p70, MCP-1, RANTES, KC, and IP-10 in the plasma measured by multiplex assay at 6 h postadministration. (B) Changes in rectal temperature before and 6 h after administration measured by a rectal temperature probe. (C) Changes in body weight before and 24 h after administration. *P < 0.05, **P < 0.01, ***P < 0.001, ****P < 0.0001 vs control as determined by two-way ANOVA and Tukey’s multiple comparison test.

### Correlation between Protein Expression, Immune Responses, and Adverse Reactions

Based on the results obtained in this study, we performed a correlation coefficient analysis between protein expression in each organ, immune responses, and adverse reactions. The analysis revealed a strong correlation between FLuc expression in the spleen and the production of S-specific IgG, the induction of S-specific CD8^+^ T cells, and fever (Figures 15A,B and S5). In addition, a positive correlation was observed between spleen FLuc expression and body weight changes (Figure 15B). FLuc expression of other organs such as TA, dLN, and liver did not significantly correlate with S-specific IgG production, S-specific CD8^+^ T cell induction, fever, or body weight changes.

**Figure 15:**
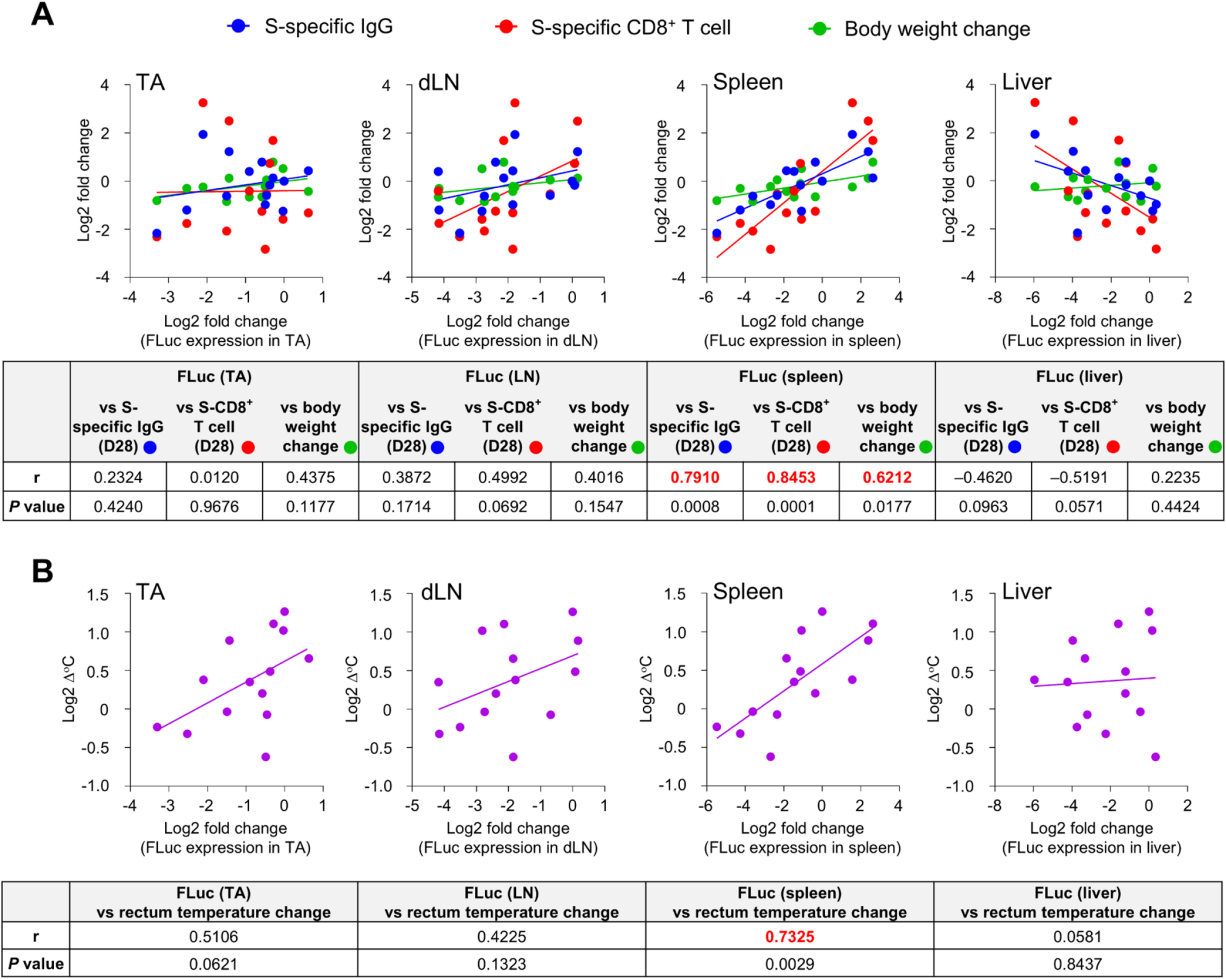
Correlation between *in vivo* protein expression and immune responses and adverse reactions. (A) Correlation scatter plot examining the relationships between protein expression in each organ (log 2 fold change between control LNP and modified LNPs) and S-specific IgG levels, S-specific CD8^+^ T cell number, and the body weight change (log 2 fold change between control LNP and modified LNPs). Correlation coefficients (*r*) and *P* values are shown in the table. (B) Correlation scatter plot examining the relationship between protein expression in each organ (log 2 fold change between control LNP and modified LNPs) and log 2 rectal temperature change. Since the changes in rectal temperature included values of zero and negative numbers, 1 was added to all values prior to performing the logarithmic transformation. Correlation coefficients (*r*) and *P* values are shown in the table.

Although protein expression in the spleen was found to correlate with the immune responses and adverse reactions, there was a concern that the systemic dissemination of mRNA-LNPs—and the resulting protein expression in distal organs such as the spleen—might depend on the injection volume of the mRNA-LNP formulation. To investigate this, we intramuscularly administered control FLuc-LNP at a dose of 1 μg mRNA/mouse, using varying injection volumes (5–50 μL) and evaluated FLuc activity in various organs 6 h after injection. The results showed comparable FLuc activity at the TA in all groups (Figure S6). Substantial FLuc activity was also observed in other distant organs (dLN, spleen, and liver), even when the administration volume was reduced to 5 or 30 μL (Figure S6). On the other hand, in the lymph nodes, FLuc activity was significantly lower in the 5 μL injection group compared with that in the 50 μL group (Figure S6). Similarly, in the spleen and liver, FLuc activity in the 5 μL group was significantly lower than that in the 30 and 50 μL groups (Figure S6). These findings suggest that larger injection volumes lead to increased protein expression in distal organs. Nevertheless, even when the injection volume was reduced to 5 μL, protein expression was still detected in distal organs, suggesting that a certain proportion of LNPs can enter the systemic circulation regardless of the injection volume.

## Discussion

This study showed that modifying the structure and content of PEG lipids, cholesterol, and phospholipids affects the immune responses and adverse reactions induced by the mRNA-LNP vaccines. Notably, certain conditions were identified that reduce adverse reactions while maintaining consistent immune responses. These findings highlight the importance of optimizing these three lipid components for improved LNP formulations.

This study specifically focused on identifying LNP formulations capable of reducing adverse reactions, such as fever, while maintaining immunogenicity. We successfully achieved this goal with several compositions, including DOPC-LNP and Stig-LNP. Fever in humans is generally initiated by the production of proinflammatory cytokines, such as TNF-α, IL-1, and IL-6, by immune cells in response to exogenous pyrogens. These cytokines, particularly IL-1 and IL-6, bind to their respective receptors on brain endothelial cells, where they induce the expression of cyclooxygenase-2 (COX-2), ultimately leading to the production of prostaglandin E2, a final mediator of fever. As demonstrated in Figures 3, 5, 7, and 9, we observed an increase in body temperature, as well as in TNF-α and IL-6 levels, following administration of control LNPs in mice. In addition, we have also demonstrated that neutralization of IL-6 using specific antibodies mitigates fever in mice following control LNP administration (manuscript in preparation). These findings suggest that similar to the mechanism of fever induction in humans, these inflammatory cytokines contribute to the onset of adverse reactions of mRNA-LNP vaccines.

Our findings demonstrated an inverse correlation between the molar ratio of PEG-lipids and antigen-specific antibody production and CD8^+^ T-cell responses (Figure 4D–F). Similarly, the length of the PEG chain in PEG-lipids was inversely correlated with CD8^+^ T-cell responses (Figure 2D–F), suggesting that steric hindrance suppresses the immune responses elicited by mRNA-LNP vaccines. This aligns with previous siRNA-LNPs studies, which showed decreased gene silencing efficiency with higher PEG modification levels.^30,31,40^

While previous *in vitro* studies have shown that higher PEG modification rates are associated with reduced delivery efficiency of the encapsulated cargo,^30^ our results demonstrated that the effect of PEGylation on *in vivo* protein expression differed across organs (Figures 2B,C and 4B,C). This organ-specific variability may be attributed to the architectural differences in each organ and the ability of the cells in those organs to capture or internalize LNPs with different features. For example, the spleen tends to trap larger particles compared to the liver.^41,42^ In our study, relatively large LNPs with shorter PEG chains (PEG_1k_) or lower PEG-lipid molar ratios (0% and 0.5%) exhibited higher protein expression in the spleen, whereas relatively small LNPs with higher PEGylation rates showed higher expression in the liver, likely due to these organ-specific characteristics.

In addition, cytokine production was more pronounced in the LNPs with shorter PEG chains (PEG_1k_, Figure 3A) and lower PEG-lipid ratios (0.5%, Figure 5A) than in the control LNP. Lipid components in LNPs are thought to activate Toll-like receptor 4 (TLR4) and the NLR family pyrin domain containing 3 inflammasomes, triggering inflammation.^43,44^ Therefore, minimal PEG modifications may enhance LNP uptake and promote lipid recognition by the innate immune system. No significant increase in body temperature was observed in the lower PEG modification groups compared with the control LNP group (PEG_1k_ and 0.5%, Figures 3B and 5B). This may represent the temperature limit in our mouse model and experimental procedures using a rectal probe. In contrast, in the group treated with LNP without PEG (0%-LNP), inflammatory cytokine levels were similar to those in the control LNP group (Figure 5A), whereas antigen-specific antibody production and CD8^+^ T cell induction were enhanced (Figure 4D–F). Although the reason why PEG-unmodified particles did not exacerbate the inflammatory response remains unclear, these results suggest that PEG-unmodified LNPs may enhance immune induction without increasing inflammation, making them promising candidates for mRNA-LNP vaccines.

From another perspective, the ability to evade immune responses against PEG is also considered an advantage of PEG-unmodified LNPs. mRNA vaccines, which are PEG-modified mRNA-LNP formulations, can induce anti-PEG antibodies following administration.^45–47^ Although recent studies have suggested that the presence of anti-PEG antibodies may not significantly affect the immunogenicity of mRNA vaccines,^48,49^ the impact of anti-PEG antibodies on immune responses and adverse reactions after repeated mRNA vaccine administration remains unclear. PEG-unmodified LNPs (PEG0%-LNPs) neither induce anti-PEG antibodies nor bind to pre-existing circulating anti-PEG antibodies. This characteristic may offer an advantage by potentially avoiding issues associated with the presence of anti-PEG antibodies.

Regarding cholesterol derivatives, we utilized the C24-alkylated cholesterol derivatives, stigmastanol, β-sitosterol, and campesterol. A previous study reported that LNPs formulated with C24-alkylated cholesterol derivatives exhibited a polymorphic morphology with lipid partitioning as well as multilamellarity, enhancing mRNA delivery *in vitro*.^36^ In this study, improved *in vitro* expression was confirmed. However, no increase in protein expression was observed at administration sites or other organs *in vivo* (Figure 6B,C). Similarly, the Stig, Sito, and Camp-LNP groups showed immune responses similar to those of the control LNP group (Figure 6D–F). In contrast, reduced cytokine production and adverse reactions were observed especially in the Stig and Sito-LNP groups (Figure 7B). Plant sterols and stanols, such as those used in this study, have anti-inflammatory properties.^50,51^ Previous studies have reported that compared with native cholesterol, cholesterol derivatives bearing alkyl substitutions at the C24 position exhibit anti-inflammatory activity, with longer alkyl chains correlating with stronger anti-inflammatory effects.^52,53^ Yuan et al. showed that cotreatment of LPS with either β-sitosterol or campesterol in RAW264.7 cells resulted in the suppression of inducible nitric oxide synthase (iNOS) and COX-2 expression compared with the results of LPS treatment alone.^53^ Furthermore, the anti-inflammatory effect was more pronounced in the β-sitosterol-treated group, which carries an ethyl group at the C24 position, compared with that in the campesterol-treated group, which has a methyl substitution at the same site.^53^ A similar trend was observed in our results, where the administration of LNPs containing stigmastanol or β-sitosterol, both of which have an ethyl group at the C24 position, resulted in a more pronounced suppression of cytokine production and adverse reactions compared with those of LNPs containing cholesterol or campesterol, which have no alkyl or methyl groups at the same position. These differences in molecular structures may have influenced the reactogenicity of Stig-LNP and Sito-LNP. Similarly, many cholesterol derivatives beyond those studied here possess different biological activities. For example, 7α,25-dihydroxycholesterol functions as a ligand for EBI2, guiding activated B and CD4^+^ T cells to migrate to the follicular region and promoting germinal center reactions.^54^ Substitution with cholesterol derivatives possessing distinct biological activities may be a potential approach for modulating the immunogenicity and adverse reactions of mRNA vaccines.

Regarding phospholipids, we substituted DSPC incorporated in the clinically approved vaccine with DOPC or DOPE. Similar immune responses were observed across all groups (Figure 8D–F), and reduced cytokine production and adverse reactions were observed in the group treated with phospholipid-substituted LNP (Figure 9). Both DOPC and DOPE contain unsaturated hydrophobic tails, suggesting that the saturation or unsaturation of the hydrophobic tail, rather than the type of headgroup, is related to the magnitude of the pro-inflammatory responses. Previous studies have reported that fatty acids regulate the TLR4 signaling pathway and downstream expression of COX-2.^39,55–58^ For example, in studies using RAW264.7 cells, the addition of fatty acids has been shown to differentially affect gene expression: saturated fatty acids (e.g., stearic acid) upregulate the expression of COX-2, iNOS, and IL-1α, whereas unsaturated fatty acids (e.g., oleic acid) do not enhance, and may even suppress, the expression of these genes.^38^ Therefore, LNPs containing DSPC may generate stearic acid during *in vivo* degradation, further promoting inflammation following LNP administration, whereas LNPs containing DOPC or DOPE may generate oleic acid, thereby inhibiting inflammation. Several other derivatives similar to those of cholesterol exist, including phospholipids with different head and tail structures.^59^ Further screening will help identify the most suitable phospholipids for mRNA-LNP vaccine formulations.

We designed LNP formulations in various combinations by varying the PEG-lipid molar ratio and replacing phospholipids and cholesterol (Figures 13 and 14), aiming to design “optimal-LNPs” which can enhance the immune response while reducing adverse reactions. The results suggest that LNPs optimized for PEG-lipid and phospholipid or PEG-lipid and cholesterol can effectively reduce adverse reactions; however, they do not enhance immune responses. We hypothesize that the lack of enhanced immune responses may be attributable to decreased particle stability resulting from the combined modifications. Furthermore, the formulation combining multiple modifications exhibited increased particle size compared with that of control LNPs (Figure 13A), which may have also contributed to the observed reduction in immunogenicity. These results highlight the importance of considering particle size, encapsulation efficiency, and stability as critical factors in optimizing lipid compositions.

Correlation analyses demonstrated a strong correlation between FLuc expression in the spleen and both immune responses and adverse reactions (Figure 15). As a prerequisite, previous studies in mice, rats, and nonhuman primates have already demonstrated that, even after intramuscular administration of mRNA-LNPs, a portion of the mRNA-LNPs can leak into the systemic circulation, ultimately reaching the spleen and expressing proteins.^12,60^ Moreover, as demonstrated in Figure S6, certain levels of protein expression were still observed in the spleen even when the injection volume of the FLuc-LNP was reduced (5–30 μL), suggesting that the observed protein expression in the spleen is not merely a result of leakage into the systemic circulation owing to physical pressure of the injected solution in mice. Furthermore, in humans, mRNA and lipid components of administered mRNA-LNPs have been detected in the bloodstream, peaking 1–2 days postadministration, suggesting that LNPs may also reach the spleen in humans.^61,62^ The spleen is a secondary lymphoid organ densely populated with antigen-presenting cells, such as dendritic cells, macrophages, and B cells. Therefore, antigen expression in this organ is expected to enhance immune responses.^63,64^ On the other hand, recognition of LNPs by innate immune cells, such as dendritic cells and macrophages, in the spleen could lead to cytokine production, possibly contributing to the observed adverse reactions.^63^ Moreover, the correlation observed in this study suggests that the level of protein expression in the spleen could serve as an indicator of reactogenicity, offering a valuable tool for screening low-reactogenicity LNP formulations. However, the findings of this study demonstrate only a correlation, and further investigations using more direct experimental methods are necessary to determine whether protein expression in the spleen genuinely contributes to the induction of immune responses and adverse reactions.

This study has several limitations. First, our experiments were conducted using a single mouse species, making the extrapolation of the data to humans difficult. This is particularly relevant to inflammatory responses. A previous study reported that the expression of the IL-1 receptor antagonist (IL-1Ra), a secreted anti-inflammatory cytokine that blocks IL-1 from binding to its receptor IL-1R, was significantly higher in mice than in humans.^65^ Elevated IL-1Ra levels may reduce sensitivity to inflammatory stimuli, thereby protecting mice against cytokine-induced adverse reactions. Consequently, although differences in inflammatory responses were observed across modified LNPs in our mouse model, the overall severity of the adverse reactions may have been underestimated. Careful interpretation of data from multiple animal species is essential to achieve a more accurate assessment. Second, the investigation into improving particle stability was limited. Although the stability of LNP formulations was evaluated (Figure S4), comprehensive long-term stability assessments of the LNPs were insufficient, and all the formulations used in this study were administered within 24 h of preparation. As shown in Figure S4, certain formulations—such as those without PEG (0%-LNP)—exhibited increased particle size after freeze–thaw cycles and reduced *in vitro* transfection efficiency, suggesting poor long-term stability. For clinical applications of the LNP formulations tested in this study, ensuring particle stability is crucial;^66^ therefore, further detailed evaluation is necessary.

## Conclusions

In conclusion, our study demonstrates that the immunogenicity and reactogenicity of mRNA-LNPs can be modulated through compositional and structural modifications of PEG lipids, cholesterol, and phospholipids, which have not been extensively explored previously. While further investigation into particle stability is required, our findings indicate that optimizing mRNA-LNPs can be achieved through a simpler approach without the need for novel ionizable lipid synthesis. These insights are expected to aid in developing mRNA vaccines with reduced inflammatory responses and adverse reactions while preserving efficacy.

## Methods

### Ethics Statements

All animal experiments adhered to the institutional guidelines of The University of Osaka for the ethical treatment of animals. Protocols were approved by the Animal Care and Use Committee of the Research Institute for Microbial Diseases at The University of Osaka, Japan (protocol number: BIKEN-AP-R01–15–3).

### mRNA Synthesis

Template plasmid DNA for mRNA synthesis was prepared using the Cloning Kit for mRNA Template (catalog number #6143, Takara Bio, Shiga, Japan) following the manufacturer’s protocols. For the transcription of mRNA expressing the S protein of SARS-CoV-2, template DNA encoding the S protein (Wuhan-Hu-1 strain), which has a glycine substitution at position 614 (D614G) and a GSAS substitution at the furin cleavage site (R682G, R683S, R685S), was amplified by PCR using KODone^R^ PCR Master Mix (catalog number #KMM-101, TOYOBO, Osaka, Japan). Template DNA was cloned into a Linearized Template Vector. Template plasmid DNAs were linearized with *Hind*III-HF (catalog number #R3104; New England Biolabs, Ipswich, MA). The digested DNAs were purified using phenol/chloroform/isoamyl alcohol (25:24:1). The mRNAs were synthesized with Takara IVTpro T7 mRNA Synthesis Kit (catalog number #6144, TakaraBio) following the manufacturer’s protocols where *N* ^1^-Methylpseudouridine 5′-Triphosphate (m^1^ψ) (catalog number #M3544, TCI, Tokyo, Japan) was used instead of UTP and CleanCap Reagent AG (catalog number #N-7113, TriLink, San Diego, CA) was added for capping. For firefly luciferase mRNA, the Positive Control Template (FLuc) in the kit served as the template DNA. The products were purified using LiCl precipitation and suspended in nuclease-free water. To eliminate dsRNA impurities, cellulose-based purification was conducted as previously described.^67^ Purified RNA was precipitated with 0.3 M sodium acetate and 50% (v/v) 2-propanol, dissolved in nuclease-free water, and stored at −80 °C.

### LNP Preparation

SM-102 was purchased from Cayman Chemical (catalog number #33474, Ann Arbor, MI, USA). DSPC, DOPC, DOPE, PEG_2k_-DMG, and PEG_5k_-DMG were purchased from NOF Corporation (catalog number #MC-8080, #MC-8181, #ME-8181, GM-020, and GM-050, Tokyo, Japan). PEG_1k_-DMG was purchased from PurePEG (catalog number #736424, San Diego, CA). Cholesterol was purchased from Sigma-Aldrich (catalog number #C8667, Burlington, MA). Stigmastanol was purchased from MedChemExpress (catalog number #HY-113494, Monmouth Junction, NJ), and β-sitosterol and campesterol were from Tama Seikagaku (catalog number #309–01401, #306–01391, Tokyo, Japan). LNPs were produced using a NanoAssemblr Ignite (Precision Nanosystems, Vancouver, BC, Canada). The organic phase was prepared by mixing lipids in ethanol. The lipid molar ratio of the control LNP was set at SM-102/DSPC/Cholesterol/PEG_2k_-DMG = 50:10:38.5:1.5 and modifications were made based on this composition. The aqueous phase was prepared by mixing mRNA in acetate buffer (6.25 mM, pH 5.0). The organic and aqueous phases were mixed at a 3:1 volume ratio and a total flow rate of 2 mL/min. Formulations contained mRNA and lipids at an N/P ratio (moles of amine in ionizable lipid: moles of phosphate in mRNA) of 5.5. The effluent was diluted with three volumes of MES buffer (20 mM, pH 5.5), followed by buffer exchange with Dulbecco’s phosphate-buffered saline (D-PBS) via ultrafiltration with an Amicon Ultra-15 Ultracell-PL 100 kDa (Merck, Burlington, MA). The hydrodynamic diameter, polydispersity index (PDI), and zeta potential of the mRNA-LNPs were measured by dynamic light scattering (Zetasizer Nano-ZS; Malvern Panalytical Ltd., Worcestershire, U.K.). The mRNA encapsulation efficiency (EE%) and content were quantified with Quant-it RiboGreen RNA Assay Kit (Invitrogen, Waltham, MA) following the manufacturer’s protocols.

### Mice

Female C57BL/6J mice (aged 6–7 weeks) were purchased from Oriental Yeast Co. Ltd. (Tokyo, Japan). The mice were acclimatized and housed in a room with a 12:12-h light: dark cycle (lights on, 8:00 a.m.; lights off, 8:00 p.m.) with free access to food and water. Anesthesia was induced by intraperitoneal injection of 0.3 mg/kg medetomidine hydrochloride (Nippon Zenyaku Kogyo Co., Ltd., Fukushima, Japan), 4 mg/kg midazolam (Maruishi Pharmaceutical Co., Ltd., Osaka, Japan), and 5 mg/kg butorphanol tartrate (Meiji Animal Health Co., Ltd., Kumamoto, Japan). Mice were euthanized by CO_2_ inhalation.

### Evaluation of Luciferase Expression

To evaluate protein expression in tissues, mice were intramuscularly injected with FLuc mRNA-encapsulating LNP (1 μg mRNA/5–50 μL/mouse). At 6 and 24 h postinjection, the muscle, lymph nodes, spleen, and liver were collected from the euthanized mice. Each tissue sample was homogenized in PBS using stainless steel beads and a bead crusher μT-12 (Taitec, Saitama, Japan). The supernatant was incubated with a Bright-Glo Luciferase Assay System (catalog number #E2620, Promega, Madison, WI) for 5 min, and luminescence was measured using a GloMax Discover Microplate Reader (Promega).

To evaluate protein expression in cultured cells, A549 cells (American Type Culture Collection, Manassas, VA) or HEK293 cells (American Type Culture Collection) were seeded in 96-well half-white plates (2 × 10^4^ cells/well, Greiner BIO-ONE, Kremsmunster, Austria) and incubated in Dulbecco’s Modified Eagle Medium (DMEM) containing 10% fetal bovine serum (FBS) and 1% penicillin/streptomycin at 37 °C for 24 h. After removing the growth medium from the cells, 100 μL of LNP solution diluted by Opti-MEM was added to each well (100 ng mRNA/well) and incubated at 37 °C for 24 h. Following incubation, 20 μL of the Bright-Glo Reagent (Promega) was added to each well and mixed. Luminescence was measured using a GloMax Discover Microplate Reader (Promega).

### Immunization

For intramuscular vaccination, mRNA-LNPs (1 μg mRNA/50 μL/dose) were injected into the TA muscle on days 0 and 21. Blood was collected on days 10, 14, 28, 35, 49, 77, and 105.

### Recombinant Protein

The S protein of SARS-CoV-2 (Wuhan-Hu-1 strain) was synthesized in-house. Briefly, the cDNA of the S ectodomain (amino acids 1–1208), which includes a glycine substitution at 614 (D614G), proline substitutions at 986 and 987 (K986P, V987P), and a GSAS substitution at the furin cleavage site (R682G, R683S, R685S), alongside the T4 fibritin foldon sequence (GYIPEAPRDGQAYVRKDGEWVLLSTFL) and an octahistidine tag (His-tag) at the C-terminus, was cloned into the pcDNA3.1 expression plasmid (Thermo Fisher Scientific). Subsequently, the expression vectors were transfected into Expi293F cells, per the manufacturer’s protocol (Thermo Fisher Scientific, #A14525). The S protein was purified with an AKTA Explorer chromatography system with a Ni-Sepharose HisTrap FF column (GE Healthcare, Chicago, IL, #17531901) and a Superose 6 Increase 10:300 GL column as previously described^15^ (GE Healthcare, #29091596).

### Detection of S-Specific Antibodies

S-specific IgG, IgG1, and IgG2b levels in the plasma were determined by ELISA as previously described.^68^ ELISA plates (Corning Incorporated, Corning, NY) were coated with recombinant S protein (1 μg/mL) in carbonate buffer at 4 °C overnight. Wells were washed three times using 0.05% Tween20 in PBS, and blocking was performed with 1% Block Ace solution (DS Pharma Biomedical, Osaka, Japan) for 1 h at room temperature (20–25 °C). Plasma samples were serially diluted in 0.4% Block Ace, and after another washing step, these dilutions were applied to antigen-coated plates and incubated for 2 h at room temperature. Horseradish peroxidase (HRP)-conjugated goat antimouse IgG (catalog number #1030–05, Dilution: 1:5000, Southern Biotech, Birmingham, AL), IgG1 (catalog number #1073–05, Dilution: 1:5000, Southern Biotech), and IgG2b (catalog number #1093–05, dilution: 1:5000, Southern Biotech) were used together with tetramethyl benzidine to detect S-specific antibodies. The colorimetric reaction was stopped with 2 N H_2_SO_4_ and assessed at OD_450–570nm_ on a microplate reader (PowerWave HT; Bio-Tek Instruments, Inc., Winooski, VT). Antigen-binding antibody concentrations were determined with reference to standard curves using monoclonal antibodies against the antigen (S-specific IgG, IgG1: catalog number #MAB105808-SP, R&D Systems, Minneapolis, MN, S-specific IgG2b: catalog number #MAB105806-SP, R&D Systems).

### Analysis of Neutralizing Antibody Titer

For the neutralization assay, VeroE6/TMPRSS2 cells (1.2 × 10^4^ cells/well) were seeded in 96-well half-white plates (Greiner BIO-ONE) and incubated in DMEM containing 10% FBS and 1% penicillin/streptomycin at 37 °C for 24 h. Serum samples on day 28 were heat-inactivated for 30 min at 56 °C and underwent 4-fold serial dilution. The diluted serum samples were mixed in a 1:1 ratio with pseudotyped viruses—replication-deficient vesicular stomatitis virus (VSV) bearing the S protein of SARS-CoV-2 (Wuhan-Hu-1 strain with D614G mutation)—and incubated at 37 °C for 1 h. After removing the growth medium from the cells, 50 μL of serum mixture was added to the wells and incubated at 37 °C for 48 h. Following infection, 50 μL of Bright-Glo Reagent (Promega) was added to each well and mixed. Luminescence was measured using a GloMax Discover Microplate Reader (Promega). The neutralization end point was defined as the serum fold dilution necessary for 90% and 99% inhibition of luciferase activity in comparison with virus control wells, in which cells were infected with the virus mixed with culture medium.

### Evaluating Blood S-Specific CD8^+^ T Cells in the Blood by Flow Cytometry

Biotinylated H-2K^b^/Spike_539–546_ (VNFNFNGL) monomers were obtained from the NIH Tetramer Core Facility and tetramerized with APC-streptavidin (catalog number #PJ27S-1, Agilent Technologies, Santa Clara, CA). Blood samples were collected on days 10 and 28 and heparinized to prevent coagulation. Blood (25 μL) was lysed twice using 200 μL of lysis buffer (155 mM NH_4_Cl, 10 mM Tris-HCl) to remove red blood cells. Obtained single-cell suspensions were stained with fixable viability dye eFluor 780 (Thermo Fisher Scientific), fluorescent dye conjugated antibodies, and tetramer for 15 min at 37 °C. The following antibodies were used for staining: AlexaFluor488 antimouse CD90.2 (catalog number #105315, dilution: 1:500, BioLegend, San Diego, CA), Brilliant Violet 510 antimouse CD44 (catalog number #103043, dilution: 1:200; BioLegend), and Brilliant Violet 605 antimouse CD8a (catalog number #100743, dilution: 1:200; BioLegend). Flow cytometry was performed using the Attune NxT Flow Cytometer (Thermo Fisher Scientific), and data were analyzed with FlowJo software version 10.9.0 (TreeStar, Woodburn, OR).

### Cytokine and Chemokines Production in Blood

To evaluate cytokine and chemokine production, S-LNPs (20 μg mRNA/100 μL/dose) were injected into the TA muscles of both legs of C57BL/6J mice (10 μg mRNA/50 μL per leg). Plasma samples were collected from the mice at 6 and 24 h and then at 7, 14, and 21 days after prime administration, and at 6 and 24 h and then at 7 and 14 days after boost administration. Plasma levels of IFN-α, IFN-β, IFN-γ, TNF-α, IL-1β, IL-6, IL-10, IL-12p70, MCP-1 (CCL2), RANTES (CCL5), KC (CXCL1), and IP-10 (CXCL10) were determined using the LEGENDplex Mouse Anti-Virus Response Panel (BioLegend, San Diego, CA), according to the manufacturer’s instructions.

### Fever and Body Weight Evaluation

To evaluate fever, mRNA-LNPs (20 μg mRNA/100 μL/dose) were injected into the TA muscles of both legs of C57BL/6J mice (10 μg mRNA/50 μL per leg), following the measurement of rectal temperature using a rectal probe (Natsume Seisakusho, Tokyo, Japan) and body weight. At 6 and 24 h and then at 7, 14, and 21 days after prime administration, and at 6 and 24 h and then at 7 and 14 days after boost administration, rectal temperature was measured. The body weights of the mice were measured at 24 h and then at 7, 14, and 21 days after prime administration, and at 24 h and then at 7 and 14 days after boost administration. Administration and measurements were performed during the light period.

### Statistical Analyses

Statistical analyses were performed using the Prism 9 software version 9.5.1 (GraphPad Software, San Diego, CA); the statistical tests included one-way and two-way analysis of variance (ANOVA), followed by Tukey’s multiple comparison test. Data presented in graphs with logarithmic axes were analyzed after logarithmic transformation. Statistical significance was set at *P* < 0.05.

## Supplementary Material



## Notes

Gating strategies for flow cytometry, neutralization curves and NT_99_ titers of serum samples, inflammatory cytokine production induced by mRNA-LNPs at an earlier time point, stability of LNPs, correlation between *in vivo* protein expression and immune responses and adverse reactions, and the effect of administration volume on *in vivo* protein expression induced by mRNA-LNPs (PDF)

## Abbreviations

COVID-19: coronavirus disease 2019
dLN: draining lymph node
DMG: dimyristoyl glycerol
DOPC: 1,2-dioleoyl-*sn*-glycero-3-phosphocholine
DOPE: 1,2-dioleoyl-*sn*-glycero-3-phosphoethanolamine
DSPC: 1,2-distearoyl-*sn*-glycero-3-phosphocholine
EE%: encapsulation efficiency
ELISA: enzyme linked immunosorbent assay
FBS: fetal bovine serum
FDA: Food and Drug Administration
FLuc: firefly luciferase
IFN: interferon
IL: interleukin
iNOS: inducible nitric oxide synthase
IP-10: IFN-γ-inducible protein 10
KC: keratinocyte-derived cytokine
LNP: lipid nanoparticle
MCP-1: monocyte chemoattractant protein-1
mRNA: mRNA
NT: neutralization titer
PCR: polymerase chain reaction
PDI: polydispersity index
PEG: poly­(ethylene glycol)
RANTES: regulated upon activation normal T cell expressed and secreted
RSV: respiratory syncytial virus
SARS-CoV-2: severe acute respiratory syndrome coronavirus 2
S: spike protein
TA: tibialis anterior
TLR: Toll-like receptor
TNF: tumor necrosis factor.
