# Supplementary material for: Modulating Immunogenicity and Reactogenicity in mRNA-Lipid Nanoparticle Vaccines through Lipid Component Optimization
Source: ACS Nano. 2025 Jul 23;19(30):27977–8001. doi: 10.1021/acsnano.5c10648 (PMC12333428; doi:10.1021/acsnano.5c10648)
Supplement: Supplementary file 1 [file nn5c10648_si_001.pdf]

Supporting Information

Modulating immunogenicity and reactogenicity in mRNA-lipid nanoparticle vaccines through lipid component optimization

Yoshino Kawaguchi[1,2], Mari Kimura[1,3], Tatsuya Karaki[1,3], Hiroki Tanaka[4,5], Chikako Ono[5,6,7], Tatsuhiro Ishida[2], Yoshiharu Matsuura[5,6,7], Toshiro Hirai[1,5,8,9], Hidetaka Akita[4,5], Taro Shimizu[1,5,8], Yasuo Yoshioka[1,3,5,7,8,9,10,*]

[1]Vaccine Creation Group, BIKEN Innovative Vaccine Research Alliance Laboratories, Research Institute for Microbial Diseases, The University of Osaka, 3-1 Yamadaoka, Suita, Osaka 565-0871, Japan.

[2]Department of Pharmacokinetics and Biopharmaceutics, Graduate School of Biomedical Sciences, Tokushima University, 1-78-1 Sho-machi, Tokushima, Tokushima 770-8505, Japan.

[3]The Research Foundation for Microbial Diseases of Osaka University, 3-1 Yamadaoka, Suita, Osaka 565-0871, Japan.

[4]Laboratory of DDS Design and Drug Disposition, Graduate School of Pharmaceutical Sciences, Tohoku University, 6-3 Aoba, Aramaki, Aoba-ku, Sendai, Miyagi 980-8578, Japan

[5]Center for Advanced Modalities and DDS, The University of Osaka, 3-1 Yamadaoka, Suita, Osaka 565-0871, Japan.

[6]Laboratory of Virus Control, Research Institute for Microbial Diseases, The University of Osaka, 3-1 Yamadaoka, Suita, Osaka 565-0871, Japan.

[7]Center for Infectious Disease Education and Research, The University of Osaka, 3-1 Yamadaoka, Suita, Osaka 565-0871, Japan.

[8]Vaccine Creation Group, BIKEN Innovative Vaccine Research Alliance Laboratories, Institute for Open and Transdisciplinary Research Initiatives, The University of Osaka, 3-1 Yamadaoka, Suita, Osaka 565-0871, Japan.

[9]Laboratory of Nano-design for Innovative Drug Development, Graduate School of Pharmaceutical Sciences, The University of Osaka, 1-6 Yamadaoka, Suita, Osaka 565-0871, Japan.

[10]Global Center for Medical Engineering and Informatics, The University of Osaka, 3-1 Yamadaoka, Suita, Osaka 565-0871, Japan.

[*]Corresponding author

Yasuo Yoshioka, PhD

Vaccine Creation Group, BIKEN Innovative Vaccine Research Alliance Laboratories, Research Institute for Microbial Diseases, The University of Osaka

3-1 Yamadaoka, Suita, Osaka 565-0871, Japan.

Tel: +81-6-6877-4919

E-mail address: y-yoshioka@biken.osaka-u.ac.jp

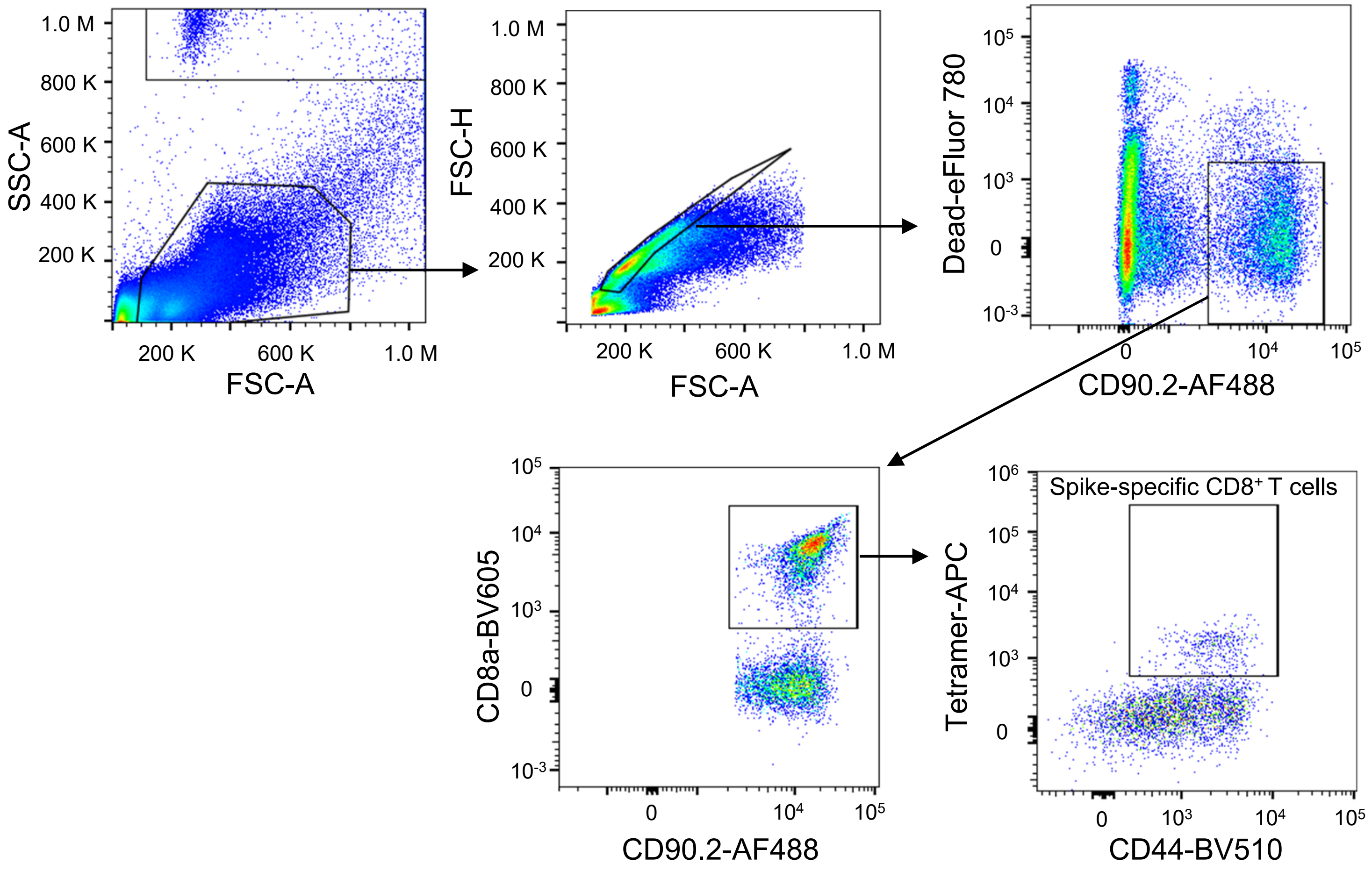

Figure S1. Gating strategy for S-specific CD8$^+$ T cells in blood after mRNA-LNP vaccination.

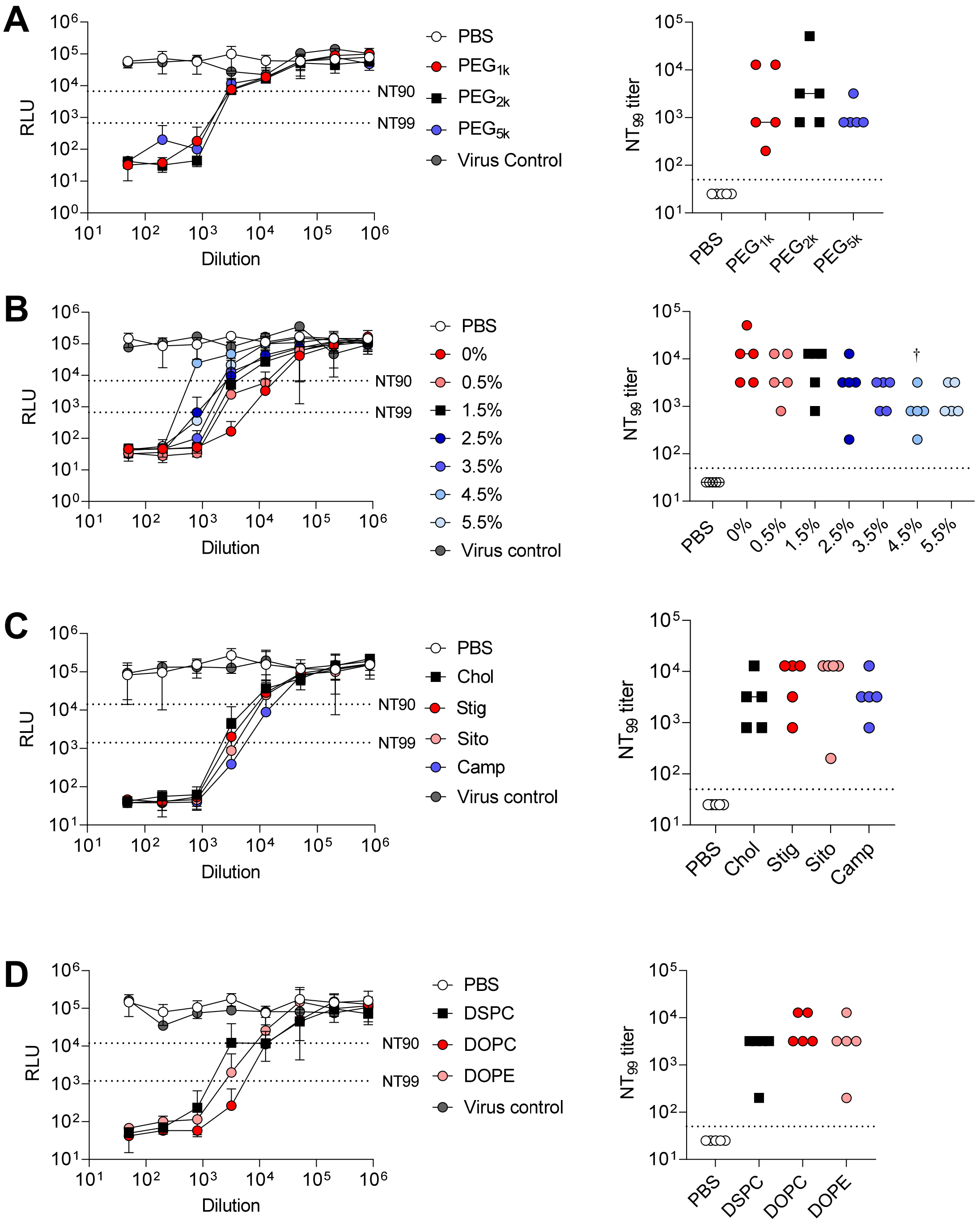

Figure S2. Neutralization curves and NT_{99} titers of serum samples. The neutralization curves obtained from the neutralization assays conducted in (A) Figure 2E, (B) Figure 4E, (C) Figure 6E, and (D) Figure 8E are presented in the left graphs. The dotted lines indicate the RLU corresponding to 90% and 99% inhibition of FLuc expression compared to when cells were infected with pseudovirus alone (Virus control). The NT_{99} titers are displayed in the right graphs.

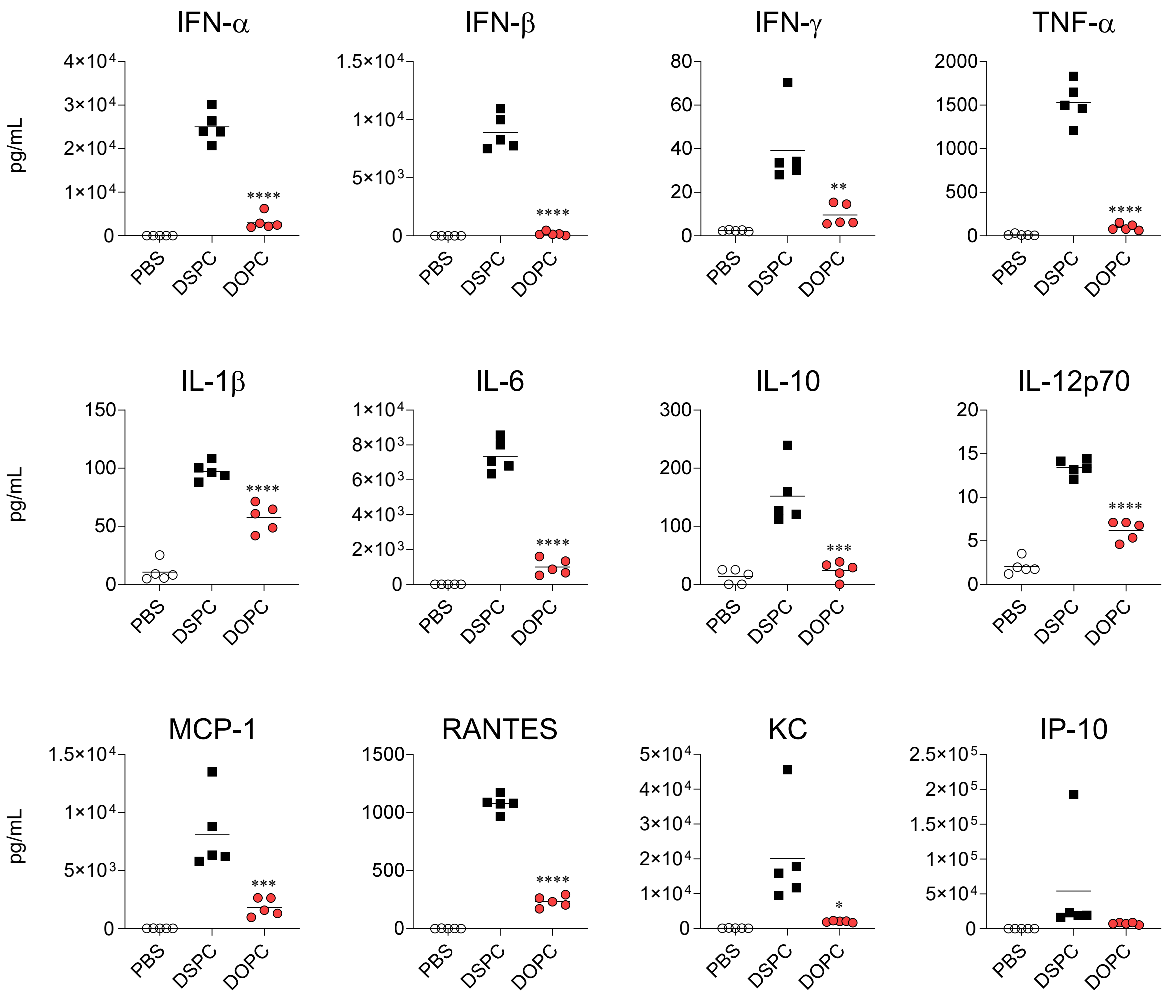

Figure S3. Inflammatory cytokine production induced by mRNA-LNPs at an earlier time point. Mice were intramuscularly administrated with S-LNPs. Concentrations of IFN-α, IFN-β, IFN-γ, TNF-α, IL-1β, IL-6, IL-10, IL-12p70, MCP-1, RANTES, KC, and IP-10 in plasma were measured by multiplex assay at 3 h post-administration. *$P < 0.05$, **$P < 0.01$, ***$P < 0.001$、 ****$P < 0.0001$ as determined by one-way ANOVA and Tukey's multiple comparisons test vs. DSPC.

A

LNP Name	Lipid component	Molar ratio
Control	SM-102 : DSPC : Cholesterol : PEG$_{2k}$-DMG	50 : 10 : 38.5 : 1.5
PEG$_{1k}$	SM-102 : DSPC : Cholesterol : PEG$_{1k}$-DMG	50 : 10 : 38.5 : 1.5
PEG0%	SM-102 : DSPC : Cholesterol : PEG$_{2k}$-DMG	50 : 10 : 40 : 0
PEG0.5%	SM-102 : DSPC : Cholesterol : PEG$_{2k}$-DMG	50 : 10 : 39.5 : 0.5
DOPC	SM-102 : DOPC : Cholesterol : PEG$_{2k}$-DMG	50 : 10 : 38.5 : 1.5
Stig	SM-102 : DSPC : Stigmastanol : PEG$_{2k}$-DMG	50 : 10 : 38.5 : 1.5

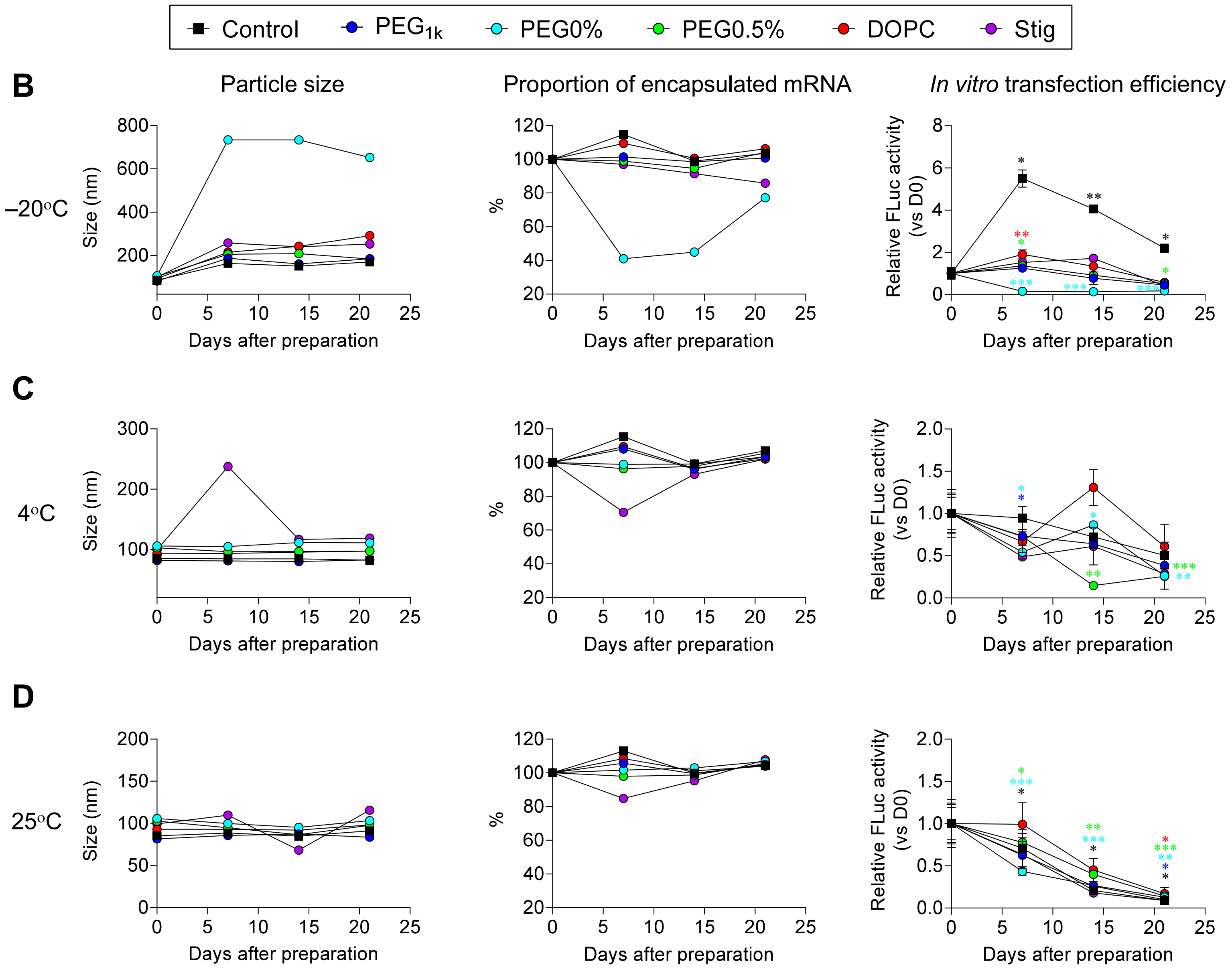

Figure S4. Stability of LNPs. (A) Lipid composition of LNPs. (B, C, D) Particle size, proportion of encapsulated mRNA, and *in vitro* transfection efficiency of LNPs with varying compositions stored at (B) −20°C, (C) 4°C, and (D) 25°C evaluated on days 0, 7, 14, and 21 after preparation. The proportion of encapsulated mRNA on day 0 was defined as 100%. To assess *in vitro* transfection efficiency, FLuc-LNPs with different compositions were transfected into HEK293 cells (100 ng mRNA/well), and the relative FLuc activity in the cell lysate was measured at each time point, normalized to the activity observed on day 0. *$P < 0.05$, **$P < 0.01$, ***$P < 0.001$ as determined by two-way ANOVA and Tukey's multiple comparisons test vs. day 0.

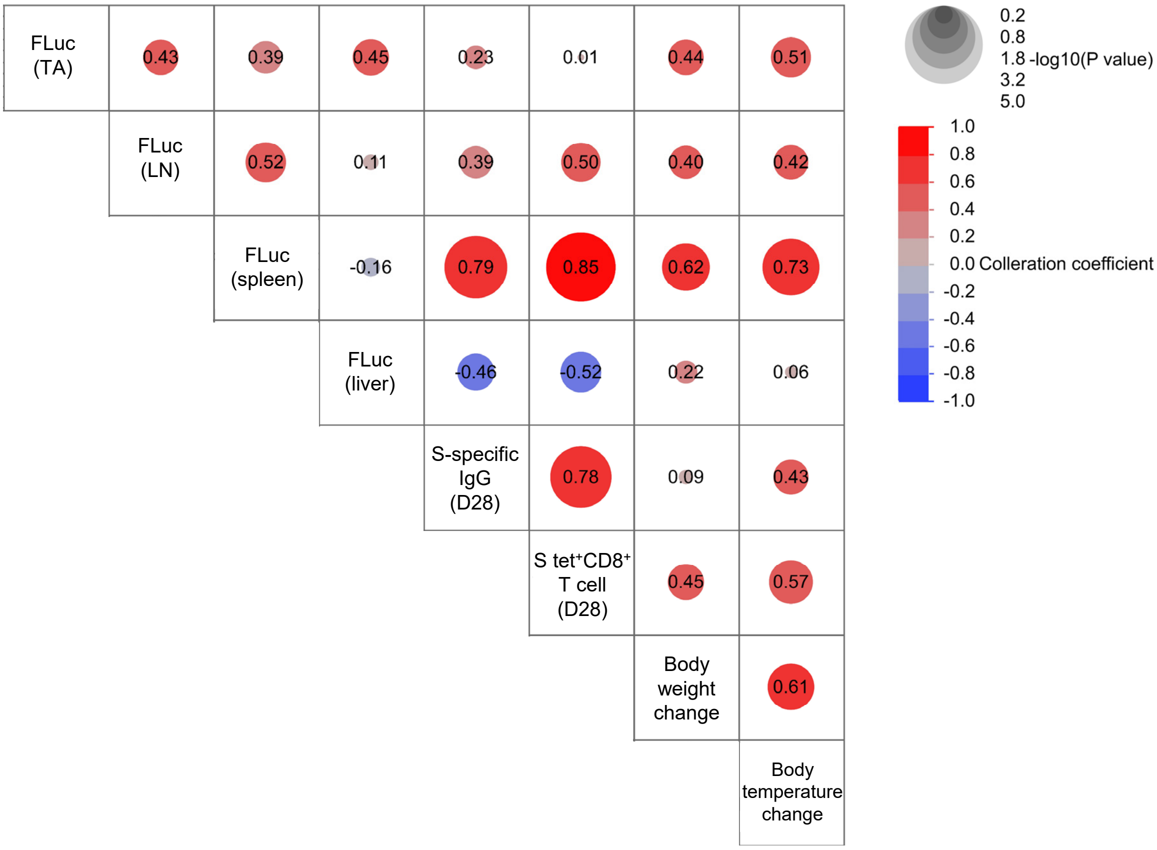

Figure S5. Correlation between *in vivo* protein expression and immune responses and adverse reactions. Correlation matrix illustrating the relationship between FLuc expression in each organ, S-specific IgG levels, S-specific CD8^{+} T cell number, body weight change, and body temperature change, created using JMP 18 software. Pearson correlation coefficients are displayed in the heatmap, with *P*-values visualized by circle size.

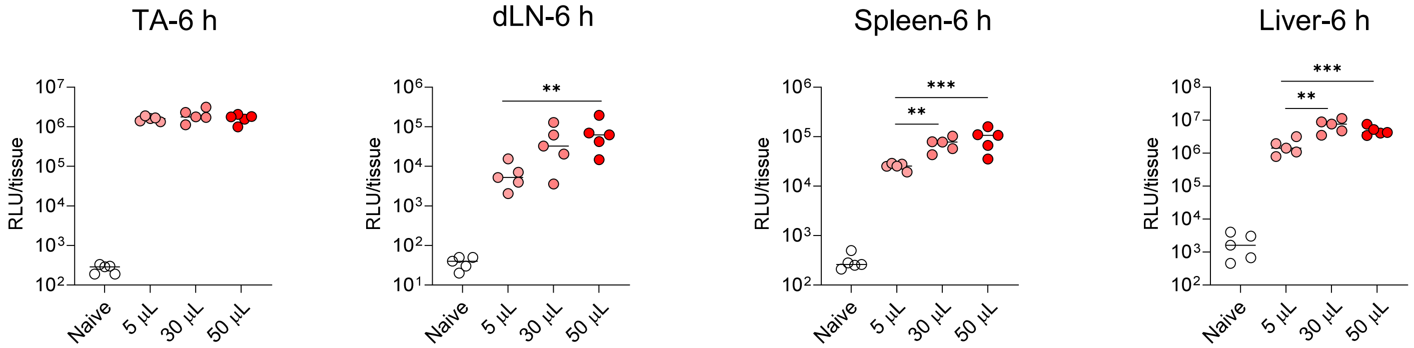

Figure S6. Effect of administration volume on *in vivo* protein expression induced by mRNA-LNPs. Mice were intramuscularly administrated with FLuc-LNPs (1 µg mRNA/5–50 µL PBS/mouse). FLuc activity in the homogenate of TA, dLN, spleen, and liver collected at 6 h post-administration was measured as relative luminescence unit (RLU). $**P < 0.01$, $***P < 0.001$ as determined by one-way ANOVA and Tukey's multiple comparisons test.
